# The effect of bio-irrigation by the polychaete *Lanice conchilega* on active denitrifiers: Distribution, diversity and composition of *nosZ* gene

**DOI:** 10.1371/journal.pone.0192391

**Published:** 2018-02-06

**Authors:** Maryam Yazdani Foshtomi, Frederik Leliaert, Sofie Derycke, Anne Willems, Magda Vincx, Jan Vanaverbeke

**Affiliations:** 1 Marine Biology Research Group, Biology Department, Ghent University, Ghent, Belgium; 2 CeMoFE, Ghent University, Ghent, Belgium; 3 Botanic Garden Meise, Meise, Belgium; 4 Aquatic Environment and Quality, Institute for Agricultural and Fisheries Research (ILVO), Ostend, Belgium; 5 Laboratory of Microbiology, Department of Biochemistry and Microbiology, Ghent University, Ghent, Belgium; 6 Marine Ecology and Management, Operational Directorate Natural Environment (OD Nature), Royal Belgian Institute of Natural Sciences, Brussels, Belgium; Auckland University of Technology, NEW ZEALAND

## Abstract

The presence of large densities of the piston-pumping polychaete *Lanice conchilega* can have important consequences for the functioning of marine sediments. It is considered both an allogenic and an autogenic ecosystem engineer, affecting spatial and temporal biogeochemical gradients (oxygen concentrations, oxygen penetration depth and nutrient concentrations) and physical properties (grain size) of marine sediments, which could affect functional properties of sediment-inhabiting microbial communities. Here we investigated whether density-dependent effects of *L*. *conchilega* affected horizontal (m-scale) and vertical (cm-scale) patterns in the distribution, diversity and composition of the typical *nosZ* gene in the active denitrifying organisms. This gene plays a major role in N_2_O reduction in coastal ecosystems as the last step completing the denitrification pathway. We showed that both vertical and horizontal composition and richness of *nosZ* gene were indeed significantly affected when large densities of the bio-irrigator were present. This could be directly related to allogenic ecosystem engineering effects on the environment, reflected in increased oxygen penetration depth and oxygen concentrations in the upper cm of the sediment in high densities of *L*. *conchilega*. A higher diversity (Shannon diversity and inverse Simpson) of *nosZ* observed in patches with high *L*. *conchilega* densities (3,185–3,440 ind. m^-2^) at deeper sediment layers could suggest a downward transport of NO_3_^−^ to deeper layers resulting from bio-irrigation as well. Hence, our results show the effect of *L*. *conchilega* bio-irrigation activity on denitrifying organisms in *L*. *conchilega* reefs.

## Introduction

Denitrification is a key process in the biogeochemical cycling of nitrogen. It is a primary loss mechanism for nitrogen in the nitrogen budget of coastal ecosystems [1]. It is a four-step respiratory process, in which nitrate (NO_3_^−^) is reduced sequentially to nitrite (NO_2_^−^), nitric oxide (NO), nitrous oxide (N_2_O), and nitrogen gas (N_2_), and is mediated by a taxonomically diverse group of microorganisms, mainly bacteria [2]. Denitrification counteracts eutrophication by removing N_2_, but it can contribute to global warming and ozone depletion due to the release of NO and N_2_O [3–5].

The distribution of denitrifying organisms and denitrification rates in marine ecosystems is affected by different environmental factors including organic matter content, concentration gradients of dissolved inorganic nitrogen (DIN: NO_3_^−^, NO_2_^−^ and NH_4_^+^) and the availability of oxygen [6,7]. Stratification and vertical distribution of denitrifying organisms also results from the vertical distribution of these environmental factors in the sediment [7,8].

Denitrifiers are facultative anaerobes and denitrification is often coupled with nitrification across oxic/anoxic interfaces in the sediment [9]. With increasing sediment depth, oxygen concentrations decrease and denitrifying communities shift from facultative anaerobes to strict anaerobes [8]. However, maximum denitrification rates do not necessarily occur at the lowest O_2_ concentration in deep layers [8,10, 11] due to low/absent NO_3_^−^ and NO_2_^−^ concentrations. The NO_x_^−^ concentration may therefore act together with O_2_ to control denitrification rates and the diversity and structure of the denitrifying community [8,10,12]. In addition, the effects of species-specific adaptation and variations in substrate threshold among denitrifying organisms need to be considered as well. For example, while oxygen has an inhibitory effect on the denitrification enzymes of *Agrobacterium tumefaciens* [13], *Pseudomonas stutzeri* is able to denitrify under aerobic conditions [14].

In general, microbial community patterns in marine ecosystems can vary from large (km or m) [15, 16, 17] to small scales (cm or less) [12,17,18]. In large scales the different patterns are due to the wide range of environmental conditions including changes in median grain size and organic matter concentration [15,16,17]. At small scales vertically in the sediment, microbial variations can be related to the different factors such as sediment types [12,19], the steepness of redox gradients [17], substrate availability [20] as well as activity and sediment mixing effects of larger fauna [17,21]. The boundary between oxic and anoxic conditions, for example, along the vertical gradient within the sediment migrates on spatial scales of micrometers to centimeters and on time scales of seconds to hours [22]. Denitrifiers have the ability to adapt to oscillations of oxygen concentrations during the rapid transition from oxic to anoxic conditions [23,24]. Such oxygen oscillations and different oxygen regimes have effects on the structure of the denitrifying community [25].

Functional diversity and abundance of larger organisms affect the biogeochemical cycles and variations in composition and diversity of microbial communities [16], creating small-scale heterogeneity in the sediment as microniches [20,26]. Building and irrigating of burrows by bioturbating organisms like worms, for example, can provide a unique environment for micro-organisms inhabiting the burrow wall [27,28] or enhance total microbial metabolism in adjacent surrounding sediments [29]. However, the impact of bioturbation depends on individual functional effects of burrowing organisms [20,28,30].

*Lanice conchilega* is a tube-building worm (Polychaeta) that glues sediment grains together to form tubes reaching 10–30 cm vertically into the sediment [31,32] and extending 2–3 cm into the water [33]. It is present throughout the North Sea and can be found in dense aggregations [34] (over 3000 ind. m^-2^ [35]), referred to as biogenic reefs [36]. This polychaete is widely distributed in soft bottom environments and acts as a bio-engineer both in intertidal and subtidal areas [37,38]. *L*. *conchilega* manifests both autogenic and allogenic ecosystem engineering properties via, respectively, its own physical structures and by transforming living/non-living materials from one physical state to another [39]. The autogenic engineering effect provides new habitat for associated species by increasing bed stability [36] and trapping organic matter from the water column [40]. It substantially affects the structure and abundance of associated communities and food-web properties [40–42]. The allogenic engineering capacity of *L*. *conchilega* is reflected in its piston-pumping activity [31] causing water and solute exchanges between tubes and the overlying water (bio-irrigation). This stimulates nutrient fluxes, mineralisation and denitrification processes in coastal sediments (as deduced from lab experiments) and seems to be density-dependent [43]. However, the effect of *L*. *conchilega* on the microbial communities mediating these processes remains unexplored.

The capacity for denitrification can be deduced from functional genes involved in denitrification pathways, which reflect the distribution and function of denitrifying organisms in the environment [1]. The detection of a gene in the environment (DNA-based methods), however, does not imply that the corresponding activity is present [44,45]. Instead, the expression of genes (RNA-based methods) and the detection of enzymes (protein-based methods) allow investigation of the direct link between the composition and density of the transcribed denitrification genes and rate of denitrification [44].

In this study, we investigated the expression of the *nosZ* gene encoding the enzyme which catalyses the final step of denitrification (conversion of N_2_O to N_2_ gas; [2]). Microbial taxa possess divergent *nosZ* clusters (typical and atypical *nosZ*) with genes that are related yet evolutionarily distinct from each other [4]. Typical *nosZ* genes, which occur more commonly in bacteria with a complete denitrification pathway (co-occurrence of denitrifying genes; [4,46]), play the major role in N_2_O reduction in coastal ecosystems [25]. Atypical *nosZ*, in contrast, seems to occur frequently in non-denitrifying organisms that do not possess any other denitrification genes and is potentially important in terrestrial environments [4,46].

Here, we investigated how distribution, diversity and composition of the typical *nosZ* gene, as well as the biogeochemical environment (oscillation in oxygen concentration, sediment grain size and chlorophyll *a*) are affected by the bio-irrigation activity of *L*. *conchilega* vertically at small (cm) scales along the sediment depth profiles and also at larger horizontal scales (m) at different natural *L*. *conchilega* densities within the reef. The observed diversity patterns in *nosZ* were then statistically linked to environmental variables to objectively assess the link between faunal-mediated heterogeneity and typical *nosZ* gene diversity.

To our knowledge, the current study is novel as most of the previous studies were limited to the bioturbation and bioirrigation impact of larger organisms on the rate of denitrification [47–51] and only a few studies have investigated this effect on the denitrifying functional genes [52–54] and still the active community investigation was neglected. As such, the current study can improve our understanding of the effects of bio-irrigation on diversity of *nosZ* gene in the active denitrifying community in marine sediments at small spatial scales.

## Materials and methods

### Study site and sampling

**Fig 1:**
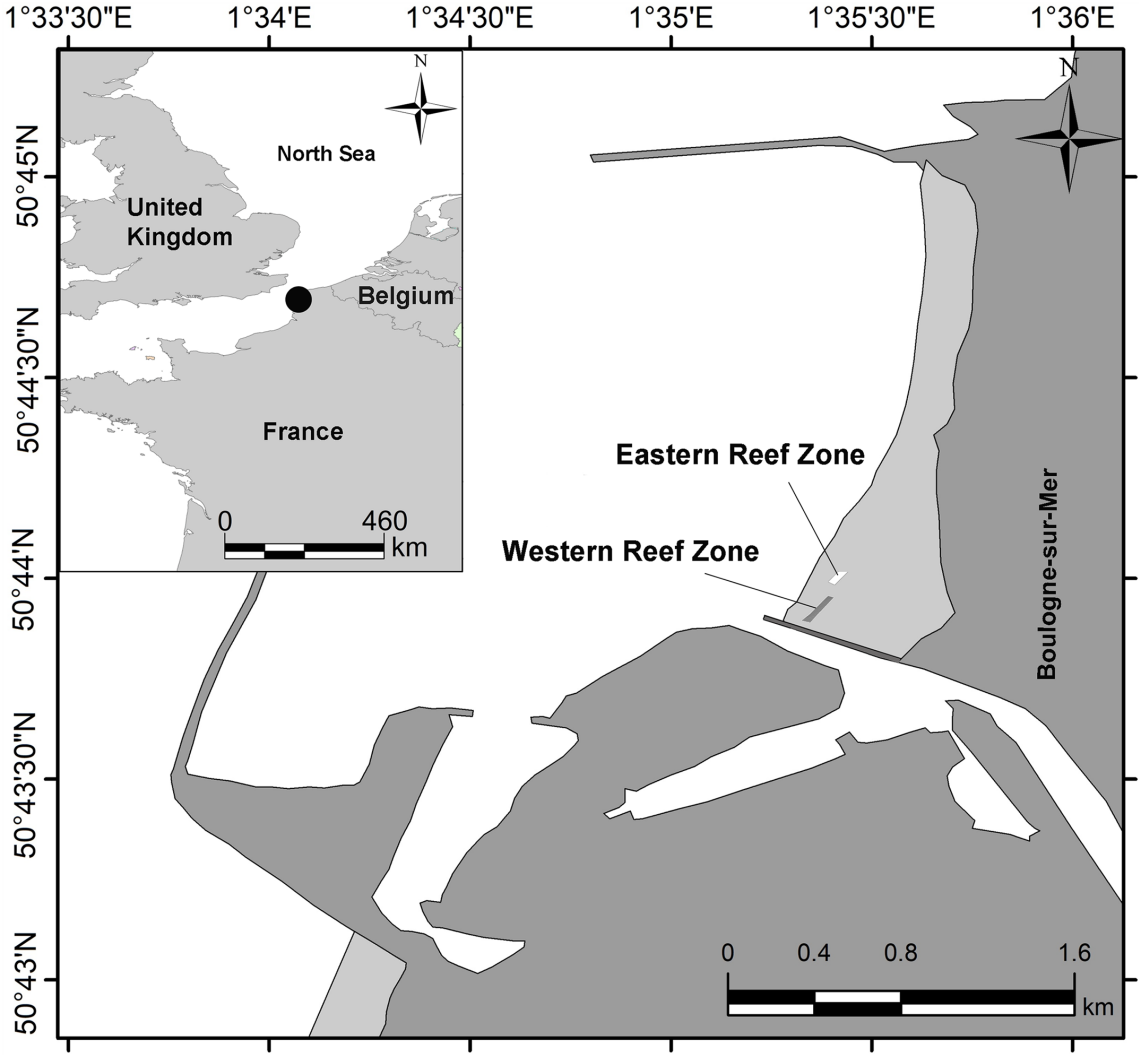
Sampling location (Western reef zone) at Boulogne-sur-Mer in France (the location of the *L*. *conchilega* reefs was obtained from Rabaut et al. [38]).

Sampling was done in October 2014 at the intertidal zone of the sandy seashore of Boulogne-sur-Mer, along the northern part of the English Channel (50° 44.01’ N, 01° 35.15’ E; Northern France; Fig 1). Sediments of the *L*. *conchilega* reef were collected by core (Plexiglas, 78.5 cm^2^ surface area; height: 25 cm) from a limited area of the western reef zone (about 4000 m^2^) located higher on the beach and exposed at every low tide [38]. No specific permits for sampling and ethics requirements were needed since our research was approved by the FWO-research proposal (G.0033.11).

Sediment samples were collected from *L*. *conchilega* patches in an area of almost 100 m^2^ within the reef. Tidal height and sediment composition were similar for all samples. Three replicate cores were taken from three different areas of the patches: *(i)* an area with an average density of *L*. *conchilega* individuals (based on counts of fringed tubes, [55]) of 25–27 tubes per core surface (3185–3440 ind. m^-2^) (hereafter referred to “high *L*. *conchilega* treatment” or “H”), *(ii)* an area with lower *L*. *conchilega* densities (5 tubes per core surface; 637 ind. m^-2^) located on the edge of the patches (“low *L*. *conchilega* treatment” or “L”), and *(iii)* sediments without *L*. *conchilega* between the reef patches (“control treatment” or “C”).

We used intact sediment cores from the reef. However, *L*. *conchilega* aggregations have been shown to support a high diversity and abundance of macrofauna in comparison with the adjacent sediments without *L*. *conchilega* [41]. Therefore, differences observed in the *nosZ* community might also result from activities of the associated community which is different from the control situation. In our sampling site, the amphipods from the genus *Urothoe* and the polychaetes *Eumida sanguinea*, *Pygospio elegans*, *Heteromastus filiformis* and *Capitella* sp. were the dominant macrofaunal species after *L*. *conchilega* [41]. Some of these species, i.e. the surficial modifier *Urothoe* and the fixed tube builder *Pygospio elegans*, are small (max 1.5 cm; [32,56,57]) and cannot affect the ecosystem as strongly as the bio-irrigator *L*. *conchilega*, making up 20–25% of total macrofaunal density in the sampling month [41]. Visual inspection did not show abundance of other large organisms in our sampling cores while *Heteromastus filiformis* and *Capitella* sp. show limited movements in the sediment [57]. *Eumida sanguinea* is a biodiffuser [57] but there is no record of the functional importance of this species for N-cycle processes. In addition, considering the high ability of *L*. *conchilega* to build reef structures [36,37], the effects on the denitrifying community in our study are therefore considered to result mainly from the presence and activity of *L*. *conchilega*.

The intact sediment cores (3 treatments × 3 replicates) were transported to the lab and submerged, uncapped without creating suspension of the sediment in tanks containing continuously aerated seawater at an *in situ* temperature-controlled room (17 °C in October). Every core was aerated separately by a stream of fine bubbles in the overlying water. In order to create water circulation inside the cores, Teflon-coated magnets were inserted at appropriate distance from the sediment surface and rotated by a central magnet in the tanks at a speed below the resuspension limit.

Within two days after sampling, vertical profiles of sediment oxygen concentration were measured (three profiles per core) in the ambient sediment at 1 to 2 cm distance from tubes, using Unisense oxygen microsensors (type ox100) in vertical increments of 250 μm. Bio-irrigation activities of *L*. *conchilega* individuals were assessed as well, by logging changes of sedimentary oxygen concentrations every second for 30–35 min at 1.5 mm sediment depth, at 1 cm distance from the tube, using the same Unisense microsensors.

At the end (three days after sampling), the upper sediment layers were sliced in 0.5 cm intervals (0–0.5 [d1], 0.5–1 [d2], 1–1.5 cm [d3]) to investigate microbial communities in the oxic and anoxic zones based on the measured oxygen profiles. This is the smallest possible scale without disturbing the sediment in the adjacent layers. A fourth layer (2.5–3 cm [d4]) was collected as well because *L*. *conchilega* was reported to ventilate its tube up to 2.4 cm tube length [31]. All sediment slices were homogenized before collecting subsamples for further analyses.

The sediment was subsampled by taking 3 to 5 mL using a cut-off syringe for the analysis of labile organic matter (chl-*a*), % clay and silt content and median grain size (MGS). Around 4 g of sediment (wet weight) was subsampled using sterilised tools for microbial analyses and stored in sterile 15-mL falcon tubes. Microbial and chl-*a* samples were immediately frozen at -80 °C, whereas the samples for grain size were dried at 60 °C before analysis.

Chl-*a* was determined by HPLC (Gilson, Middleton, Wisconsin, USA) analysis according to [58] and sediment granulometry by laser diffraction (Malvern Instruments, Malvern, UK).

### RNA extraction and *nosZ* sequencing

RNA was extracted from 4 g homogenised sediment (wet weight) from each depth layer (36 samples = 3 treatments × 3 replicates × 4 layers) using the RNA Power Soil Total RNA isolation kit (MO BIO Laboratories). Integrity and purity of RNA extractions were checked following [16]. The DNA-free RNA samples were reverse transcribed into cDNA using Omniscript Reverse Transcriptase Kit (Qiagen) according to the instructions of the manufacturer and using 10 μM of Random Hexamer primers (Applied Biosystems) and 5 μL RNA template per total volume of a reaction (20 μL).

RT-PCR amplification of *nosZ* gene fragments (267 bp) was performed in three technical replicates for each sample using specific primer sets (*nosZ*-2F: 5’-CGCRACGGCAASAAGGTSMSSGT-3’ / *nosZ*-2R: 5’-CAKRTGCAKSGCRTGGCA GAA-3’) [3] for the typical *nosZ* gene cluster [4,46]. The forward primer contained the 5’ Illumina adaptor, forward primer pad and linker and the *nosZ*-2F primer. The reverse primer consisted of the reverse complement of the 3’ Illumina adapter, 12-bp multiplex identifiers (MIDs), the reverse primer pad and linker and the *nosZ*-2R primer. MIDs in the reverse primer were used to identify the different samples (see S1 Table for adaptor, MID, pad, linker and primer sequences for the forward and reverse data; [59]). A control (no template) was also included to ensure that no contamination occurred in the laboratory.

Amplifications were performed in volumes of 25 μL containing 5 μL of target cDNA, 5 μL of 5xKAPA HiFi Buffer (containing 2 mM MgCl_2_ at 1x; Kapa Biosystems, Boston, Massachusetts, United States), 0.4 μg/μL BSA, 0.2 mM dNTPs, 0.4 μM of each primer, and 1.5 U KAPA HiFi HotStart DNA Polymerase (1 U/μL) (Kapa Biosystems). The ‘touchdown’ PCR conditions were as follows: after an initial denaturation step at 95 °C for 5 min, 20 cycles were performed consisting of three steps: denaturation (98 °C, 20 sec), annealing (70 °C, 1 min, decreasing 0.5 °C cycle^-1^ to 60 °C) and extension (72 °C, 1 min) followed by 15 additional cycles in which the annealing temperature was 60 °C [60]. The final elongation step was performed at 72 °C for 10 min.

PCR amplicons of the three technical replicates of each sample were combined, purified using E-Gel (Invitrogen, Life technologies) and measured with a Qubit fluorometer (Life Technologies). Samples were then pooled in equimolar amounts and loaded on a Bioanalyzer 2100 (Agilent Technologies) to check the presence of the single peak. Purified pooled libraries were submitted to Genomics Core (Center for Human Genetics UZ—K.U. Leuven) for 150×2-cycle paired-end sequencing on an Illumina Miseq platform.

### Sequence analyses

Demultiplexing of the data was carried out by Genomics Core using the Illumina standard procedure by applying the bcl2fastq tool of Illumina which allows for 1 mismatch between the barcodes. Raw demultiplexed Illumina fastq files were then quality-filtered and merged using Pear v0.9.5 (Paired-End read mergeR; [61]). Reads shorter than 100 bp or longer than 400 bp and low-quality reads (scores <25) were removed from the output. Any reads containing uncalled bases and singletons were also discarded. The final file was checked for quality control with Fastqc v0.11.3 (http://www.bioinformatics.babraham.ac.uk/projects/fastqc/) [62].

We eliminated potential chimeras using the USEARCH package [63,64]. Non-target reads (non-*nosZ* reads) were filtered out using FrameBot included in the Fungene pipeline (http://fungene.cme.msu.edu/). We retrieved a reference set of *nosZ* protein sequences of 163 different species with high scores (>919) and 98% coverage with a hidden Markov model (HMM) from Fungene. FrameBot compared each member of these reference sequences to the query nucleotide sequence in both forward and reverse directions. In addition, FrameBot corrected insertion and deletion errors, and translated DNA sequences to frameshift-corrected protein sequences [64,65].

*nosZ* diversity was determined at the amino acid level. Therefore, a complete identity threshold (100%) of amino acid sequences was applied in USEARCH. These units (unique AA *nosZ* sequences) were considered to be operational taxonomic units (OTUs).

Prior to further analysis, OTUs with <0.005% relative abundance in all samples, which together accounted for less than 2% of the total reads and mostly represented singletons and doubletons, were discarded [66,67].

Maximum likelihood phylogeny of unique *nosZ* AA sequences was inferred using RAxML v8.2.6 on the CIPRES Science Gateway [68], under a JTT+I+G model as determined using the lowest AIC criterion in ProtTest version 2.4 [69]. Node confidence was determined using 200 bootstrap replicates. In order to construct a phylogeny with reference sequences, we performed protein BLAST searches against the NCBI non-redundant protein database (http://blast.ncbi.nlm.nih.gov/Blast.cgi) of 25 OTU representatives selected randomly across the phylogenetic tree (S2 Fig). For each blastp search, the best 100 blast hits were retained. To reduce the size of the tree, identical sequences were removed as well as sequences differing in 1 or 2 AA positions. A final alignment of 208 *nosZ* sequences from the database was used to construct a ML tree using RAxML as described above.

### Data analyses

#### Environmental data analyses

We used permutational multivariate ANOVA (PERMANOVA; Primer v6.1.10., Primer-E Ltd., Plymouth, United Kingdom with the PERMANOVA + add-on package; [70]) to perform a two-way fixed factor model design with “treatment” and “depth” as factors. “Replicate” was nested as a random factor in “treatment” [71]. We investigated whether significant differences in environmental factors (MGS, % silt+clay [mud] content and chl-*a* concentration) among treatments and at different depths were observed. To test for the differences in maximum oxygen penetration depth (max OPD) and oxygen profiles (both depth and time profiles) between levels of *L*. *conchilega* density, PERMANOVA with a one-way fixed factor model design (“Treatment” as a factor) was performed. Prior to the PERMANOVA analyses for oxygen profiles, a multivariate data matrix was constructed in which each depth horizon (for depth profiles) and each second (for time profiles) was considered a “variable” [72]. Pairwise tests were performed for significant terms using Monte Carlo p-values when the number of possible permutations was restricted [70].

Euclidean distance was used to calculate the resemblance matrices in PERMANOVA for the univariate environmental data [70] and also for the multivariate data matrix corresponding to oxygen depth and time profiles [72]. Homogeneity of multivariate dispersion (‘variance’) was tested with PERMDISP for any of the significant terms in PERMANOVA to verify any dispersion effects on the significant results from the PERMANOVA tests. For oxygen depth profiles, SIMPER analysis was used to determine which depth was responsible for the identified differences.

#### Microbial community analyses and diversity estimates

To correct for technical bias related to read number variations among the samples, the data was normalised [73]. All samples were normalised by taking the proportions of each OTU, multiplying it with the minimum sample size (1022 reads) and rounding to the nearest integer [74,75].

To calculate alpha diversity and to assess how the sampled community of *nosZ* genes reflects a “true” diversity per sample [76], we made random sub-samples of the non-normalised data matrix to the minimum number of reads (1022) in R version 3.3.1. This was repeated 1000 times and the average diversity (richness, Shannon-Wiener [log e] and inverse Simpson) of each sample was calculated from the estimated diversity of each trial.

Rarefaction curves were also calculated in R for the number of observed OTUs vs. the total number of reads to investigate the variation of OTU numbers in our samples. Generalised UniFrac distances (α = 0.5) [77] were calculated on the composition of *nosZ* from normalised data with the GUniFrac package in R [78]. PERMANOVA was conducted on these UniFrac distances using the Adonis package in R. PERMDISP and pairwise difference tests were also performed in R.

Euclidean distance was used to calculate the resemblance matrices in PERMANOVA (Primer v6.1.10) for the diversity indices (estimated as average values).

PERMANOVA was performed as a two-way fixed factor model design (“treatment” and “depth” as factors while “replicate” was nested in “treatment”) to test significant differences among factors in composition and diversity indices of *nosZ*.

Principal coordinates analysis (PCoA) plots were generated using the Ade4 package in R to visualize variation in composition of *nosZ* among treatments and depths.

In addition, we investigated whether differences among factors were caused by differences in non-abundant OTUs (<1% relative abundance), by constructing a dataset with abundant OTUs (defined as OTUs with >1% relative abundance in at least one treatment-depth combination). This resulted in a second dataset with 21 abundant OTUs out of the total 502 OTUs and with 439624 reads. Statistical analyses on the dataset with the abundant OTUs were performed as described above.

Multivariate data corresponding to *nosZ* sequences were reduced to binary data (OTUs were counted as present or absent) in Excel [79]. Jaccard similarity index and Venn diagrams were calculated and drawn based on the number of shared and unique OTUs among treatments per depth layer [79,80].

### Multiple linear regressions

Multiple linear regressions were performed to identify the variables contributing significantly to the observed variations in the diversity indices of *nosZ* (richness, Shannon diversity and Inverse Simpson as response variables) in our study. Multivariate data related to oxygen profiles, both depth and time profiles, were also included in the regression analyses but as two univariate variables: mean values of oxygen concentration per depth layer (“OX”) and coefficient of oxygen variations over time (“CV”), respectively. To avoid multi-collinearity among independent variables, the regression analyses were performed after removing highly correlated variables based on *(i)* the correlation coefficient (lrl ≥ 0.85) among numerical independent variables, and *(ii)* graphical exploratory techniques (boxplots) displaying the distribution of each numerical variable at different depths and treatments (categorical independent variables). No collinearity was observed among numerical variables while “depth” and “treatment” were collinear with chl-*a* and max OPD, respectively. Six explanatory variables were ultimately entered into the models: chl-*a* concentration, MGS, % silt+clay [mud] content, CV, max OPD and OX. All assumptions [81] were checked after model selection. An initial linear regression analysis showed violation of homogeneity of residuals in all models. We therefore applied linear regression with generalised least-squares (GLS) using restricted maximum-likelihood (REML) estimation. This incorporates variance–covariates to model the variance structure. To find the optimal random structure, the full linear regression model was compared to the GLS models of specific variance structures using lowest Akaike information criteria (AIC). This procedure resulted in the use of a variance structure that allowed for different variances per stratum for “max OPD” (varIdent function, R package nlme). The optimal fixed components in the final model were obtained by applying a backward selection using the likelihood ratio test obtained by ML estimation. A graphical model validation was applied to check for homogeneity and normality in the final models [82].

## Results

### Sediment environmental factors

The granulometric variables, including MGS and mud content (%), were not significantly influenced by interactive and single effects of treatments (high, low and no (= control) *L*. *conchilega*) and depths (PERMANOVA, p > 0.05). chl-*a* concentration was significantly affected by depth (PERMANOVA, pseudo-F = 12.455, p = 0.000). Highest chl-*a* concentrations were observed at the top layer (0–0.5 cm) (pairwise test, all 0.001 < p < 0.01) followed by the second layer which was significantly higher than the deepest layer (2.5–3 cm) (pairwise test, p = 0.014; Table 1).

**Table 1:** Variations of sedimentary environmental factors in three *L*. *conchilega* treatments and four depths.

	*Chlorophyll a (μg g*^*-1*^*)*			*MGS (μm)*			*Mud content (%)*		
	High	Low	Control	High	Low	Control	High	Low	Control
Depth (cm)									
**0–0.5**	2.01±0.28^A^	1.55±0.06^A^	1.80±0.20^A^	238.12±6.35^A^	227.14±2.96^A^	243.23±6.22^A^	1.55±1.55^A^	0.20±0.20^A^	0.00±0.00^A^
**0.5–1**	1.34.±0.19^B^	0.79±0.23^B^	1.50±0.06^B^	250.97±4.14^A^	228.81±4.96^A^	245.96±5.62^A^	2.17±2.17^A^	0.28±0.28^A^	0.15±0.15^A^
**1–1.5**	0.65±0.15^BC^	1.04±0.55^BC^	1.29±0.12^BC^	239.25±6.43^A^	232.76±6.11^A^	242.12±3.36^A^	0.00±0.00^A^	0.00±0.00^A^	1.46±1.11^A^
**2.5–3**	0.59±0.51^C^	0.56±0.13 ^C^	1.02±0.49 ^C^	235.31±8.97^A^	238.03±7.71^A^	234.62±1.39^A^	2.94±2.94^A^	0.00±0.00^A^	1.31±1.31^A^

Treatments: high and low *L*. *conchilega* densities and control; depths: 0–0.5, 0.5–1, 1–1.5 and 2.5–3 cm. Capital letters stand for significant vertical differences (A > B > C) across treatments (p > 0.05). MGS: Median grain size

**Fig 2:**
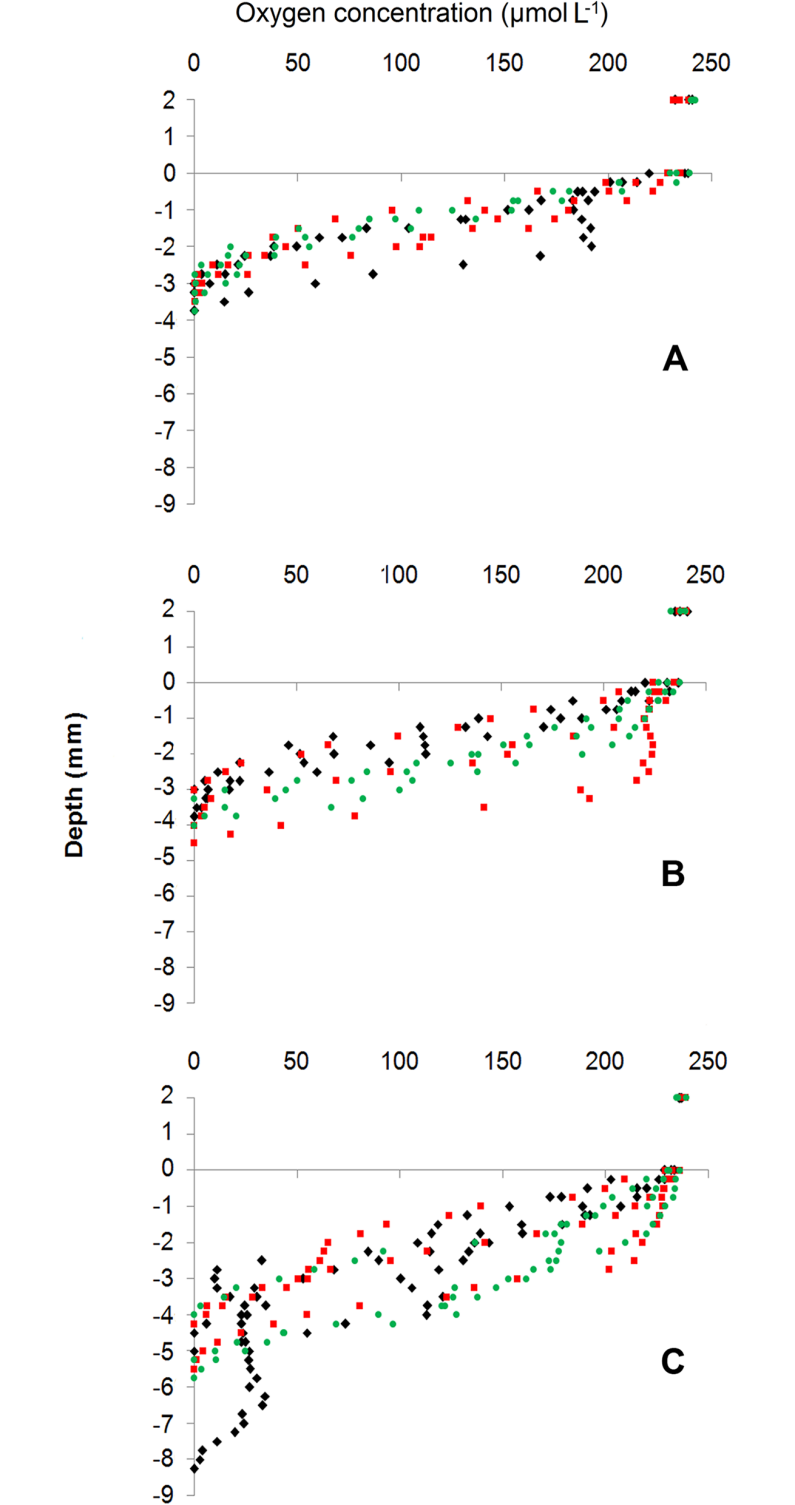
Oxygen depth profiles in the overlying water and sediment. Control (A), low (B) and high *L*. *conchilega* (C) treatments. Nine profiles were measured per treatment (shown with different symbols and colours per core replicate). The zero depth in the profiles represents the sediment–water interface.

Oxygen depth profiles (Fig 2) were significantly affected by the treatment effect (PERMANOVA, pseudo-F = 5.33, p = 0.006) (S2 Table). Pair-wise tests (S2 Table) revealed significant differences in vertical oxygen distribution between control and both *L*. *conchilega* treatments (p = 0.002 and p = 0.039 for high and low *L*. *conchilega* density, respectively). There were no significant differences between both *L*. *conchilega* treatments. SIMPER analysis showed that the difference in oxygen profiles between the control treatment and high *L*. *conchilega* treatment was attributed to 1–4 mm sediment depth whereas this difference was between 1–3.25 mm for the control and low *L*. *conchilega* treatments.

**Fig 3:**
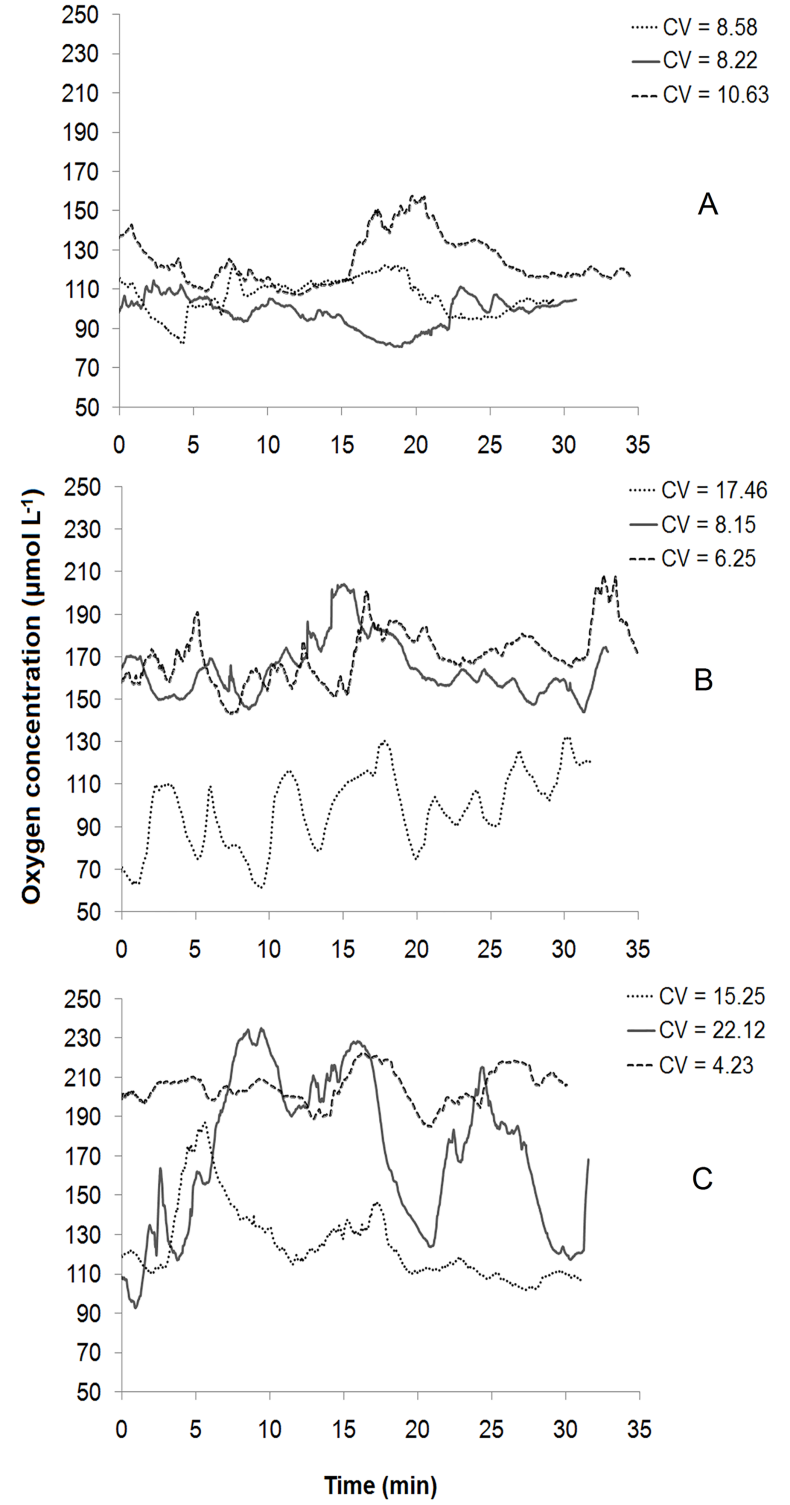
Time variations of oxygen concentration as a measure for bio-irrigation activities of *L*. *conchilega*. Measured in 30–35 min at 1.5 mm depth in control (A), low (B) and high *L*. *conchilega* (C) treatments. Three profiles were measured per treatment. “CV” indicates coefficient of oxygen variations over time.

Oxygen concentrations over time (over a period of 30–35 min) measured at 1 cm distance from the tube and at 1.5 mm sediment depth remained high in the high *L*. *conchilega* (93–235 μmol L^-1^) and in the low *L*. *conchilega* treatment (60–203 μmol L^-1^). The lowest values were observed in the control treatment (80–157 μmol L^-1^) (Fig 3). However, oxygen time profiles were not significantly affected by treatment (p > 0.05; S2 Table).

Maximum OPD was significantly affected by treatment (pseudo-F = 12.19; p = 0.000) and pair-wise tests showed a significantly higher max OPD in the high *L*. *conchilega* density treatment (5.17 ± 0.44 mm; mean ± se) compared to the low (3.69 ± 0.17 mm; p_(MC)_ = 0.008) and control (3.30 ± 0.12 mm; p_(MC)_ = 0.001) treatments (S2 Table; Fig 2).

PERMDISP analyses showed some heterogeneity of multivariate dispersion (F = 3.16, p = 0.028) in the analysis of maximum OPD but this was not the case for the analysis of the entire oxygen depth profiles (p > 0.05).

### Composition of *nosZ* and diversity indices

In total, 545,742 *nosZ* reads were assigned to 502 OTUs. The current sampling effort was sufficient in most samples to reflect the variation of the observed OTUs (Fig A in S1 Fig). Only samples of the Hd4 treatment in all three replicates (H1d4, H2d4 and H3d4) did not reach an asymptotic rarefaction curve. Instead, read numbers of the high *L*. *conchilega* treatment decreased with increasing depth (lowest read numbers in Hd4; see S3 Table). This pattern was consistent across replicate samples, suggesting that the low read numbers are not a technical artifact but probably a reflection of the lower read numbers of OTU in this treatment.

**Fig 4:**
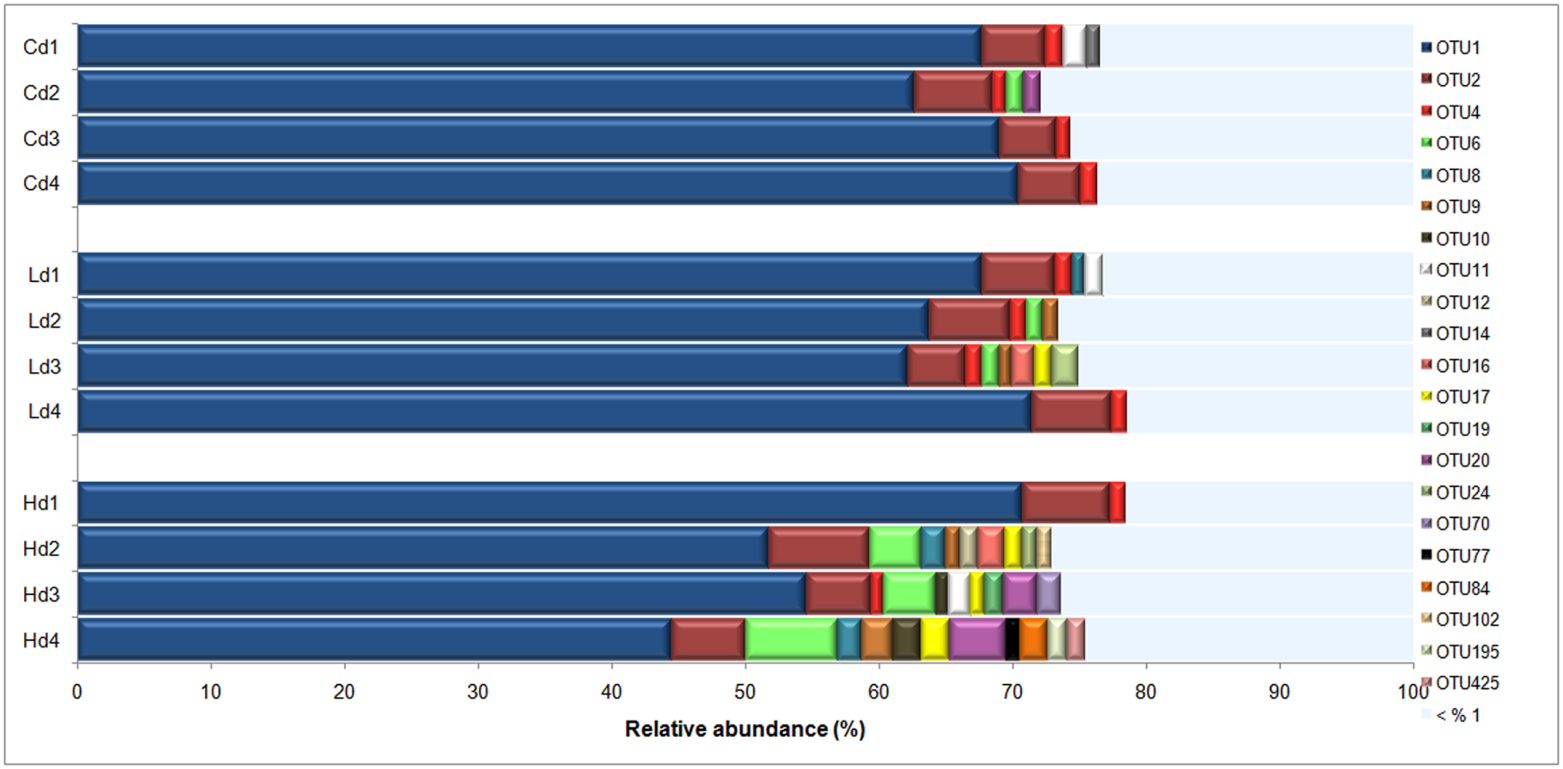
Distribution of abundant and non-abundant OTUs at different depths and treatments. Abundant OTUs were defined as OTUs with >1% relative abundance in at least one treatment-depth combination (values are averages of replicates) and non-abundant OTUs defined as OTUs with <1%. “H” indicates high *L*. *conchilega* treatment. “L”: low *L*. *conchilega* treatment, “C”: control treatment, “d1”: 0–0.5 cm depth, “d2”: 0.5–1 cm, “d3”: 1–1.5 cm, “d4”: 2.5–3 cm.

Only 21 of 502 OTUs were found to be abundant (>1% relative abundance in at least one treatment-depth combination). Of these abundant OTUs, the two dominant OTUs 1 and 2 combined constituted 50–77% of the community where OTU 1 alone accounted for 44–71% of the relative abundance in different samples (Fig 4).

Numbers of reads and total numbers of OTUs per sample as well as the numbers of abundant OTUs (>1% relative abundance) in treatment-depth combinations are provided in S3 Table.

The results of the protein blast indicate that among the abundant OTUs, OTUs 1, 2, 4 and 10 were most closely (>97% AA identity) related to *nosZ* sequences from *Pseudomonas stutzeri* constituting 76% of the community. The other abundant OTUs were less than 95% similar to protein sequences available in sequence databases. In addition, a blast search was performed of all 502 nosZ OTUs against NCBI’s non-redundant protein database (nr) with an e-value threshold of 1E-20 and taking into account only the first blast hit for each OTU. These results showed that 380 OTUs (which corresponds to 93.3% of all reads) were most closely related to *Pseudomonas stutzeri*.

The maximum likelihood tree of *nosZ* sequences retrieved from our samples showed considerable phylogenetic diversity (S2 Fig). No clear phylogenetic signal could be observed with respect to treatment, i.e. samples from the three treatments (control, low and high *L*. *conchilega*) contained sequences from across the phylogenetic tree (S2 Fig). Phylogenetic analysis of representative OTUs together with related sequences derived from blastp searches showed that there were three major clusters belonging to the Alpha, Beta and Gamma classes of the Proteobacteria and abundant as well as non-abundant OTUs are dispersed among these clusters (S3 Fig).

PERMANOVA based on Generalized Unifrac distance showed that the composition of *nosZ* gene with and without including non-abundant OTUs was significantly affected by the single effect of treatment and depth, regardless of the dataset used (Table 2).

**Table 2:** Results from PERMANOVA analysis main tests for differences in composition of *nosZ* gene.

	*factor*	*df*_*term*_	*Pseudo-F*	*P value*
***All OTUs***	treatment	2	2.82	**0.026**
	depth	3	2.68	**0.012**
***Abundant (> 1%) OTUs***	treatment	2	3.35	**0.025**
	depth	3	2.59	**0.046**

Treatments: high and low *L*. *conchilega* densities and control; depths: 0–0.5, 0.5–1, 1–1.5 and 2.5–3 cm. Analyses were carried out based on Generalized UniFrac distances (α = 0.5) on the data sets using all OTUs or only abundant OTUs with relative abundance >1%. Single factor results are shown while the interaction treatment × depth is not significant.

Pairwise tests (S4 Table) indicated that the composition of *nosZ* in the treatment with high density of *L*. *conchilega* was significantly different from the control treatment (p = 0.036). However, no difference in *nosZ* gene composition was observed between the low and high *L*. *conchilega* treatments, and also between low *L*. *conchilega* and control treatments (p > 0.05).

In the vertical depth distribution, pairwise tests revealed that the composition of *nosZ* at the top layer (0–0.5 cm) was significantly different from the other layers (all pairwise test p < 0.05; S4 Table).

**Fig 5:**
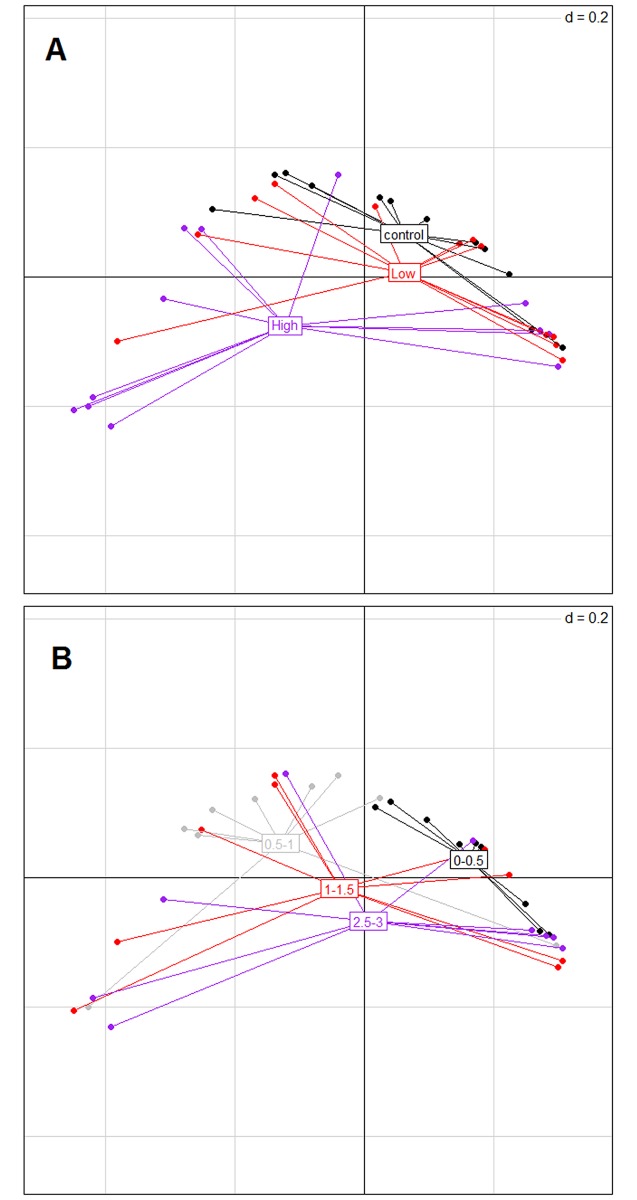
PCoA based on generalized UniFrac distances on the normalised data including all OTUs. A: per treatment; B: per depth. Each point represents a sample. Three treatments (High: high *L*. *conchilega* treatment, Low: low *L*. *conchilega* treatment, Control) and four depth layers (0–0.5, 0.5–1, 1–1.5 and 2.5–3 cm) are shown.

The highly significant PERMDISP results in both analyses with all OTUs (factor “treatment”: F = 10.07, p = 0.000; factor “depth”: F = 8.95, p = 0.000) and only abundant OTUs (factor “treatment”: F = 10.93, p = 0.000; factor “depth”: F = 7.56, p = 0.000) indicated a dispersion effect as well. Visualization by PCoA plot (Fig 5 and S4 Fig) showed that dispersion increased with increasing *L*. *conchilega* densities and with increasing depth in the sediment.

**Fig 6:**
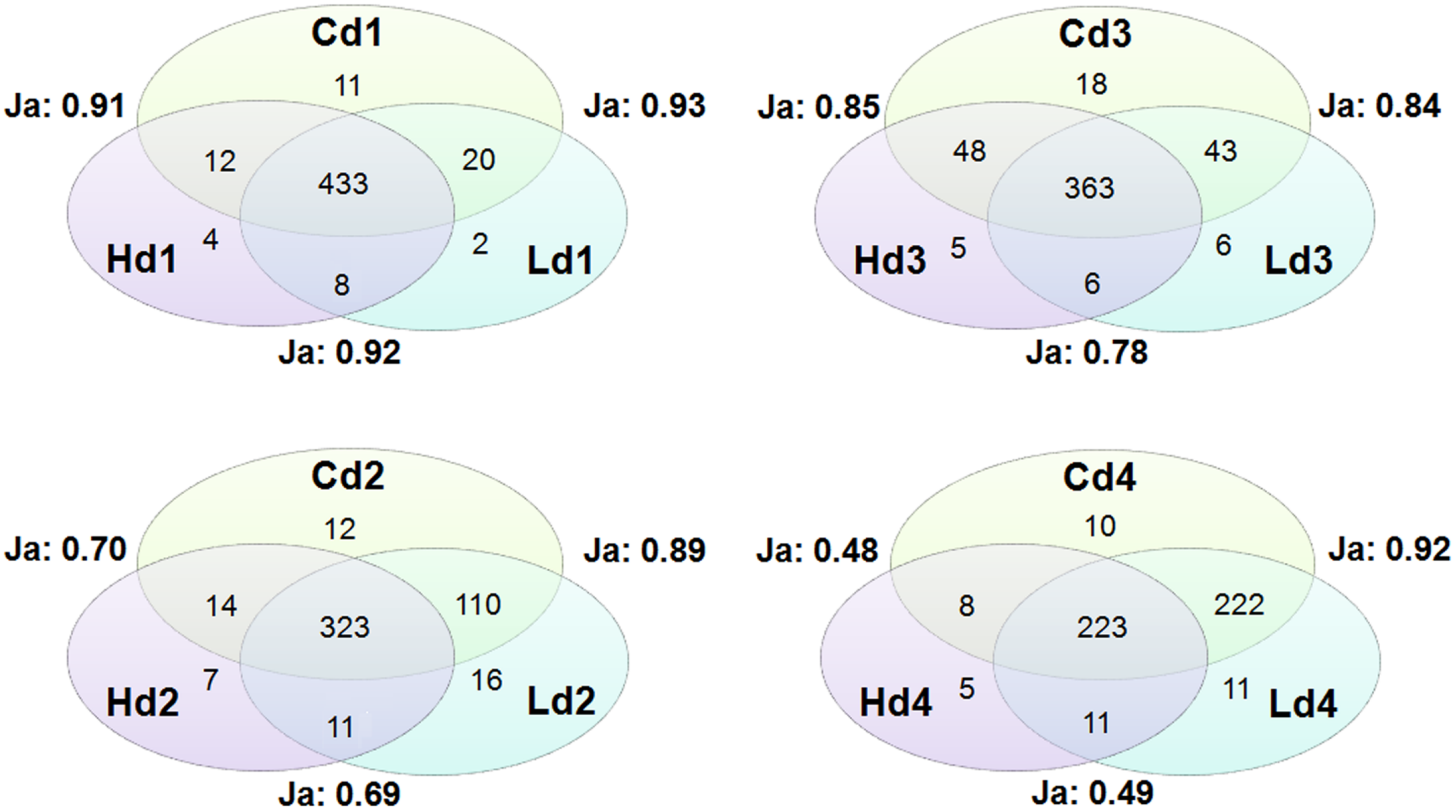
Venn diagrams of the numbers of unique and shared OTUs and Jaccard similarity index (Ja). Treatments: high *L*. *conchilega* treatment (H), low *L*. *conchilega* treatment (L), Control (C); depth: 0–0.5 (d1), 0.5–1 (d2), 1–1.5 (d3) and 2.5–3 cm (d4).

The Venn diagram and Jaccard similarity index (Fig 6) indicated the highest similarity in OTUs among treatments at the top layer (0–0.5 cm) with more than 91% similarity. With increasing depth, the number of shared OTUs was generally reduced between the high *L*. *conchilega* density and the two other treatments, reaching the lowest similarity (<50%) at the deepest sediment layer (2.5–3 cm). However, 231 out of 247 (93.5%) and 234 out of 247 (94.7%) of OTUs in Hd4 were shared with Cd4 and Ld4, respectively (Fig 6). Among different depths per treatment, the lowest value (47%) of the Jaccard similarity index was observed when comparing the top layer (0–0.5 cm) and the 2.5–3 cm layer of the high *L*. *conchilega* treatment (Table 3).

**Table 3:** Jaccard similarity index in each treatment between the top layer (0–0.5 cm) and other depth layers.

*High L*. *conchilega*	Jaccard	*Low L*. *conchilega*	Jaccard	*Control*	Jaccard
Hd1-Hd2	0.67	Ld1-Ld2	0.89	Cd1-Cd2	0.91
Hd1-Hd3	0.82	Ld1-Ld3	0.81	Cd1-Cd3	0.93
Hd1-Hd4	0.47	Ld1-Ld4	0.89	Cd1-Cd4	0.92

Treatments: high *L*. *conchilega* treatment (H), low *L*. *conchilega* treatment (L), Control (C). Depths: 0–0.5 (d1), 0.5–1 (d2), 1–1.5 (d3) and 2.5–3 cm (d4)

**Fig 7:**
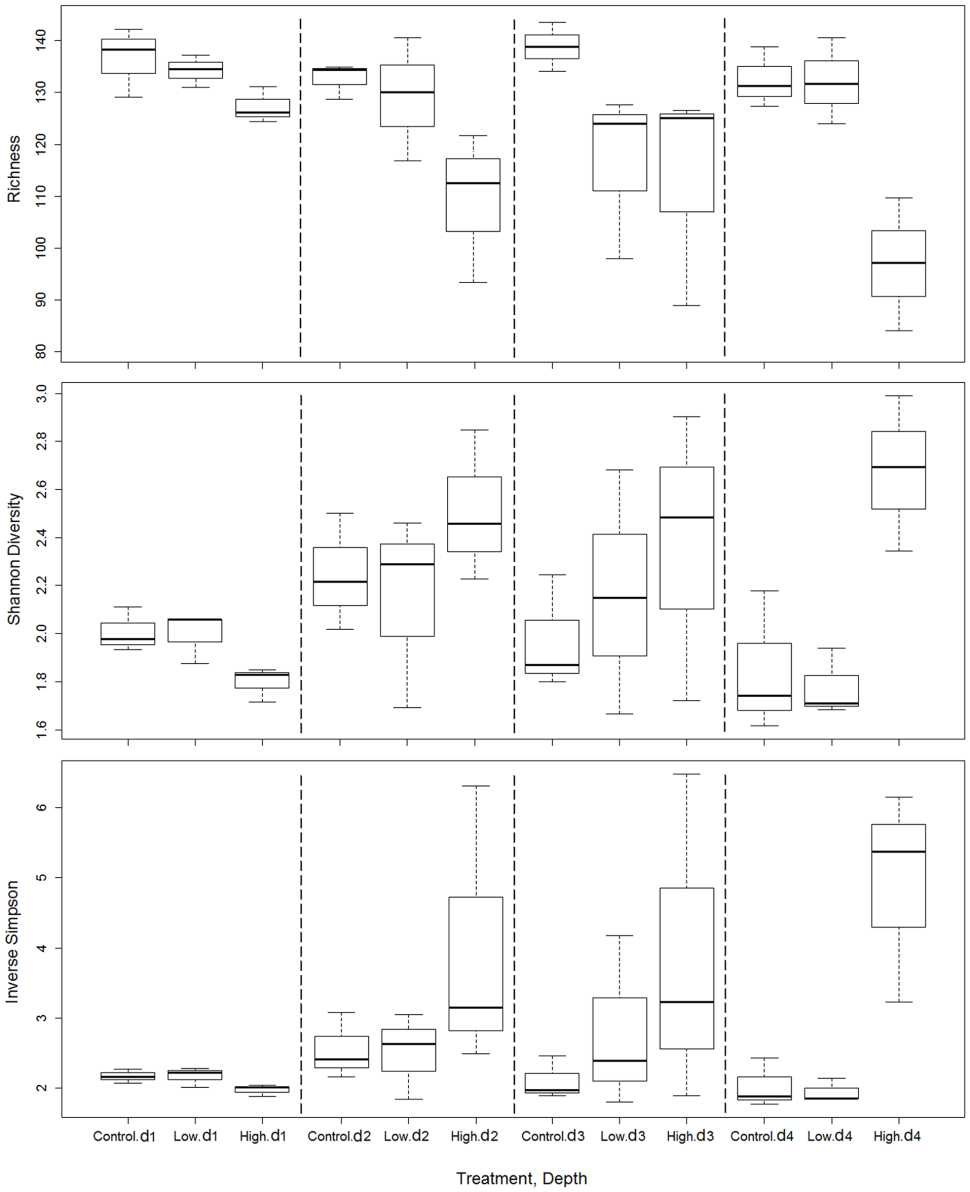
Vertical distribution of diversity indices (richness, Shannon diversity and inverse Simpson) of *nosZ* gene. Obtained from 1000 sub-samples of the data matrix to the minimum number of reads at three treatments (high *L*. *conchilega*, low *L*. *conchilega* and control treatments) and four depth layers (0–0.5 [d1], 0.5–1 [d2], 1–1.5 [d3] and 2.5–3 [d4] cm). The solid horizontal line shows the median. Box is drawn between the quartiles (the middle 50% of the data). Dotted lines extend to the minimum and maximum values.

PERMANOVA did not show any significant effect on Shannon diversity and inverse Simpson for the factors “depth”, “treatment” or the interaction (all p > 0.05) although higher values were observed in the high *L*. *conchilega* treatment at highest depth (S5 Table; Fig 7). On the other hand, OTU richness was affected by the interaction effect “treatment × depth” (PERMANOVA, pseudo-F = 5.45, p = 0.043; S5 Table). Pairwise tests (S6 Table; Fig 7) did not reveal significant differences in OTU richness among treatments at the top layer (0–0.5 cm) (p_(MC)_ > 0.05). In deeper sediment layers (2.5–3 cm), lower values were detected in the high *L*. *conchilega* treatment (pairwise tests, p_(MC)_ = 0.015 and p_(MC)_ = 0.016 compared with the control and low *L*. *conchilega* treatments). In addition, richness was significantly lower in the high *L*. *conchilega* compared to the control at the depth 0.5–1 cm (pairwise test, p_(MC)_ = 0.026).

We generally found a decreasing trend in OTU richness in the high *L*. *conchilega* treatment with increasing depth (Fig 7). These changes were significant between the top (0–0.5 cm) and deepest (2.5–3 cm) layer (pairwise test, p_(MC)_ = 0.022; S6 Table).

### Statistical modeling to link abiotic sediment characteristics with the diversity indices of *nosZ* gene in *L*. *conchilega* reefs

Maximum oxygen penetration depth (max OPD; collinear with “treatment”) was an important variable incorporated in all diversity models. However, it was not significant in the model for the inverse Simpson index (*p* > 0.05). We found significant negative relation (β = -8.10x10^-3^, *p* < 0.01) between max OPD and OTU richness; and a positive relation between this variable and Shannon diversity (β = 1.96 × 10^−4^, *p <* 0.01) (Table 4). The average oxygen concentrations per depth layer (OX) and % mud content were further important explanatory variables in the model of Shannon diversity. Shannon diversity was negatively related with both OX (β = -3.19 × 10^−3^) and % mud content (β = -0.06). Apart from max OPD, chl-*a* concentration contributed significantly (β = 7.24, *p <* 0.001) to the model of richness.

**Table 4:** The average values of diversity indices of *nosZ* gene as a function of environmental variables.

Model	formula
Richness	S = 154.40*** − 8.10 e^-03^ max OPD** + 7.24 Chl-*a* ***
Shannon diversity	H’ = 1.45*** − 0.06 Mud* + 1.96e^-04^ max OPD** − 3.19e^-03^ OX *
Inverse Simpson	1/λ = 1.47*** + 2.1e^-04^ max OPD

Richness (S), Shannon diversity (H’), inverse Simpson (1/λ), maximum oxygen penetration depth (max OPD), average of oxygen concentrations per depth layer (OX), % mud content (Mud) and chlorophyll *a* (Chl-*a*); Significance levels are indicated with *(p < 0.05), **(p < 0.01), or ***(p < 0.001).

## Discussion

In the current study, we investigated the denitrifying community in *L*. *conchilega* aggregations at the intertidal zone by focusing on expression of the gene encoding nitrous-oxide reductase (*nosZ*), the enzyme catalysing the final step of denitrification. We investigated typical *nosZ* genes, which play the major role in N_2_O reduction in coastal ecosystems [25]. The denitrifiers with a complete pathway were identified as the dominant community [25].

In comparison with previous studies on functional genes for denitrification (*nirS*, *nirK* and *nosZ*) in marine sediments [12,18,83,84], our results show a diverse active *nosZ* community (502 OTUs) in the *L*. *conchilega* reef (area of *L*. *conchilega* aggregations plus zones without *L*. *conchilega* as a control). This is what can be expected in intertidal sediments where regular oxygen oscillations support a high genetic potential for denitrification [25].

The highest phylogenetic diversity was found in the Alphaproteobacteria and a high percentage of the sequences were closely related to *Pseudomonas stutzeri* (class Gammaproteobacteria). This is in agreement with previous studies in shelf sediments [25,85] and shallow coastal sediments [12,86,87]. In addition, the high relative abundance of Gammaproteobacteria-related phylotypes in our RNA-derived libraries suggests high levels of metabolic activity within this group [86].

### Horizontal and vertical patterns of the active *nosZ* community in *L*. *conchilega* aggregations

Our study revealed differences in the composition of *nosZ* at both horizontal (m) and vertical (cm) scales between high *L*. *conchilega* and control treatments, and between top oxic (0–0.5 cm) and deeper layers. These differences in composition were mainly due to changes in the relative contributions of abundant OTUs to the community. The same pattern was also observed in the *nosZ* community structure in the high *L*. *conchilega* treatment at a depth of 2.5–3 cm (Fig 6). Statistical modelling of diversity measures suggested that biogeochemical heterogeneity (oxygen concentrations and oxygen penetration depth) created by the activity of high *L*. *conchilega* aggregations is the driving factor behind the observed differences, next to chl-*a* and mud content (%) (Table 4). This suggests a structuring effect of *L*. *conchilega* on the expression of *nosZ* genes in our study site. However, MGS and % mud were not affected by treatment nor depth, and chl-*a* was only affected by depth in the sediment. This is in agreement with the previous study in the same sampling location in Boulogne-sur-Mer and at the same sampling month in October [41]. However, temporal fluctuations in *L*. *conchilega* population density can significantly affect environmental properties [88]. Therefore, autogenic effects of *L*. *conchilega* on the denitrifying communities cannot be completely excluded. In our case study, however, there was a mainly allogenic effect of *L*. *conchilega* on the environment.

### Allogenic effect of *L*. *conchilega*

*Lanice conchilega* bio-irrigates its tube through piston-pumping activity for 1.5 min generally every 4 min, thereby transporting about 3 mmol O_2_ m^-2^ d^-1^ into the sediment [31]. *Lanice conchilega* introduces oxygen-rich water in layers where oxygen is absent [31]. This intermittent ventilation results in temporal changes in oxygen concentrations in the upper layers of the sediment [31] and a deeper OPD especially close to the *L*. *conchilega* tubes [31,43]. Our results reveal a density-dependent effect of *L*. *conchilega* bio-irrigation on the OPD (Fig 2) as well as a significantly higher oxygen content at the top sediment layer (mainly 1–4 mm) in high and low *L*. *conchilega* treatments compared to the control sediment (Fig 2). In addition, the periodic piston-pumping activity resulted in oxygen variations over time. The oscillations were generally higher in both *L*. *conchilega* treatments compared to the control treatment (Fig 3), although there is no statistically significant difference. This could be caused by the fact that for some measurements, variations in oxygen concentration (the values of CV) were comparable with those in the control treatment. This can be explained by merging pulses from piston pumping activity of adjacent animals or by merging individual pulses in one animal resulting from a high frequency of occurrence of this pumping activity [31].

Generally low richness and high diversity (Shannon and inverse Simpson) of *nosZ* was observed in the high *L*. *conchilega* treatment compared with the control treatment at depth with most prominent differences at the deepest sediment layer (2.5–3 cm). At this depth, a significant difference between the high and low *L*. *conchilega* treatments was also observed. In a vertical sediment depth profile, nitrate coexists with oxygen in the top layer. In the deeper layers where oxygen is low or absent, the nitrate concentration is an important factor determining similarity or difference in anaerobic denitrifying communities [89,90]. As anoxic conditions continue at depth, nitrate concentrations decrease [8] while denitrification progresses. This trend coincides with the decrease in diversity of denitrifying organisms and the rate of their activity with increasing sediment depth [8,91,92]. The differences observed in diversity indices of *nosZ* between treatments at depth may thus be explained by the different availability of NO_3_^−^ between treatments in the low oxygen or anaerobic deeper layers. The higher availability of NO_3_^−^ in the high *L*. *conchilega* treatment can be by directly pumping NO_3_^−^ [47] and/or by bringing O_2_ to deeper layers [31] increasing the surface for coupled nitrification–denitrification in the sediment along the tube [29] and also by more intensive irrigation activity in this treatment, compared to the low *L*. *conchilega* and control treatments. The most prominent differences between the high *L*. *conchilega* and the other two treatments were at the deepest sediment layer (2.5–3 cm). These differences are not due to adaptation of certain OTUs specific to the high *L*. *conchilega* treatment: 93.5% and 94.7% of OTUs in Hd4 were shared with Cd4 and Ld4, respectively (Fig 6). Instead, observed differences between treatments are due to reduction in the number of non-abundant and rare OTUs in Hd4 compared with Cd4 and Ld4.

*Lanice conchilega* ventilates its tube in each pumping activity by a volume of water equivalent to 2.4 cm tube lengths [31]. Therefore, the most suitable condition for denitrification (concomitant high availability of NO_3_^−^ and little or no oxygen in the surrounding sediment) was met at 2.5–3 cm sediment. In the upper layers, high richness values resulted from the presence of a larger number of non-abundant and rare OTUs mainly belonging to the Alphaproteobacteria. This highly diverse pool may provide a background reservoir for suboptimal denitrification conditions increasing niche creation [90,93,94]. In addition, considering denitrification is an anaerobic process, the presence of active *nosZ* community in the top oxic layer in all treatments (Fig 7) may point to co-consuming oxygen and NO_3_^−^ as electron acceptors (aerobic denitrification [10]) by denitrifying organisms in the sediment of our study area. This process has also been observed before in marine sediments [10].

Finally, the higher variations in composition of *nosZ* in the *L*. *conchilega* treatments compared with the control (Fig 5A) are in accordance with a previous study indicating higher variations in denitrification rates in bioturbated sediment [51]. In bioturbated sediments, nitrogen cycle processes are most likely driven by the formation of microniches surrounding the burrow systems [51]. In our study, this pattern can also be visualised in “depth” with increasing depth (Fig 5B).

## Conclusions

This study improves our understanding of the effects of bio-irrigation on the *nosZ* gene. Our results reveal that large densities of *L*. *conchilega* (>3000 ind. m^-2^) affect composition, structure and diversity indices of the active *nosZ* community both vertically and horizontally in the sediment. These patterns can be linked with heterogeneity in geochemical environments in the sediment that are affected by allogenic ecosystem engineering effects of *L*. *conchilega*. The piston-pumping activity of the polychaete transports oxygen rich waters to the deeper layers of the sediment. This could also provide higher availability of NO_3_^−^ at depth by increasing the surface for coupled nitrification-denitrification in the sediment along the tube.

Since *L*. *conchilega* is present throughout the North Sea, often in dense aggregations, our results provide evidence for the implementation of this polychaete in marine Ecosystem-Based Management. This study illustrates that a future loss of macrobenthic diversity, and especially ecosystem engineers, will have important repercussions for benthic ecosystem functioning.

## Supporting information

S1 Fig Rarefaction curves of the *nosZ* gene sequeces. Plotting the number of observed OTUs (unique AA *nosZ* sequences) as function of the number of sequences screened in samples with >4000 reads (A) and ≤4000 reads (B). “H” indicates high *L*. *conchilega* treatment. “L”: low *L*. *conchilega* treatment, “C”: control treatment, “d1”: 0–0.5 cm depth, “d2”: 0.5–1 cm, “d3”: 1–1.5 cm, “d4”: 2.5–3 cm.
(TIF)

S2 Fig Maximum likelihood phylogeny of *nosZ* amino acid sequences retrieved from our samples. 10% of OTUs (50 OTUs out of 502) making up around 84% of the total reads are shown. Abundant OTUs (>1% relative abundance in at least one treatment–depth combination) are indicated in red. The heat map (on the right) illustrates the average relative abundance of each OTU per sample. The dominant OTU 1 is shown in red. Numbers at nodes are bootstrap values (values <60% not shown). The scale bar represents 10% sequence divergence (10 mutations per 100 sequence positions).
(TIF)

S3 Fig Maximum likelihood phylogeny of *nosZ* amino acid sequences retrieved from our samples and reference sequences. To construct a phylogeny with reference sequences, protein BLAST searches of 25 OTU representatives selected randomly across the phylogenetic tree in S2 Fig were performed against the NCBI non-redundant protein database. Abundant OTUs (>1% relative abundance) are shown in red. Numbers at nodes are bootstrap values (values <50% not shown). The scale bar represents 30% sequence divergence (30 mutations per 100 sequence positions).
(TIF)

S4 Fig PCoA based on generalized UniFrac distances on the normalised data including only abundant OTUs. A: per treatment; B: per depth. Each point represents a sample. Three treatments (High: high *L*. *conchilega* treatment, Low: low *L*. *conchilega* treatment, Control) and four depth layers (0–0.5, 0.5–1, 1–1.5 and 2.5–3 cm).
(TIF)

S1 Table Primers for paired-end *nosZ* sequencing on the Illumina Miseq platform. Adaptor, Multiple Identifier (MID), pad, linker and primer sequences for the forward and reverse data sets are given.
(DOCX)

S2 Table Results from PERMANOVA analysis for differences in oxygen concentrations (depth and time profiles and max OPD) among treatments. Treatments: high and low *L*. *conchilega* densities and control. Analyses were based on Euclidean distance similarity matrix. Where the treatment effect was significant, pairwise tests were performed within treatments. P-values were obtained by permutation and drawn from Monte-Carlo samplings (p_(MC)_) if the number of unique permutations was less than 100 [70]. Max OPD indicates maximum oxygen penetration depth. Bold values indicate significant differences at p < 0.05.
(DOCX)

S3 Table Numbers of reads and total numbers of OTUs per sample as well as the numbers of abundant OTUs (>1% relative abundance) in treatment-depth combinations are provided. (DOCX)

S4 Table Results from PERMANOVA analysis pairwise tests for differences in composition of *nosZ* influenced by the single effects of “treatment” and “depth”. Analyses were carried out based on Generalized UniFrac distances (α = 0.5) on the data sets using all OTUs or only abundant OTUs with relative abundance >1%. Treatments: high and low *L*. *conchilega* densities and control; Depths: 0–0.5, 0.5–1, 1–1.5 and 2.5–3 cm.
(DOCX)

S5 Table Results from PERMANOVA analysis main tests for differences in diversity indices of *nosZ* among treatments and depths. Treatments: high and low *L*. *conchilega* densities and control; depths: 0–0.5, 0.5–1, 1–1.5 and 2.5–3 cm. Diversity indices (richness, Shannon-Wiener [log e] and inverse Simpson) were calculated from the average values obtained from 1000 sub-samples of the data matrix to the minimum number of reads (1022). Univariate analyses of diversity indices were based on Euclidean distance similarity matrix. P-values obtained by permutation.
(DOCX)

S6 Table Results from PERMANOVA analysis pairwise tests for differences in OTU richness influenced by the interaction effect “treatment × depth”. Three treatments (High: high *L*. *conchilega* treatment, Low: low *L*. *conchilega* treatment, Control) and four depths (0–0.5, 0.5–1, 1–1.5 and 2.5–3 cm). Richness was calculated from the average values obtained from 1000 sub-samples of the data matrix to the minimum number of reads (1022). Analyses were based on Euclidean distance similarity matrix. P-values obtained by permutation.
(DOCX)
