# Supplementary figures and images for: The effect of bio-irrigation by the polychaete Lanice conchilega on active denitrifiers: Distribution, diversity and composition of nosZ gene
Source: PLoS One. 2018 Feb 6;13(2):e0192391. doi: 10.1371/journal.pone.0192391 (PMC5800672; doi:10.1371/journal.pone.0192391)

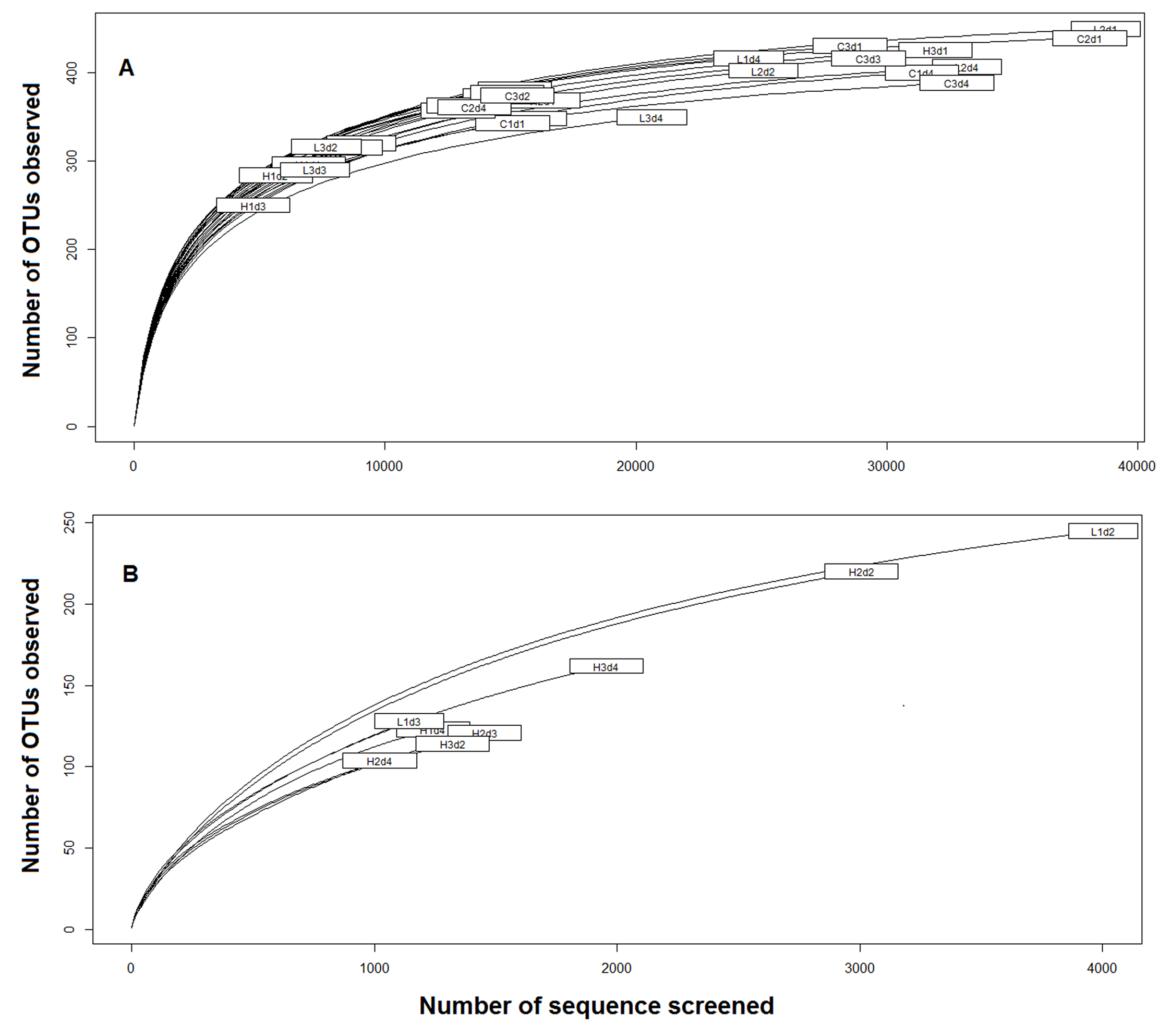

Supplement: S1 Fig — Plotting the number of observed OTUs (unique AA nosZ sequences) as function of the number of sequences screened in samples with >4000 reads (A) and ≤4000 reads (B). “H” indicates high L. conchilega treatment. “L”: low L. conchilega treatment, “C”: control treatment, “d1”: 0–0.5 cm depth, “d2”: 0.5–1 cm, “d3”: 1–1.5 cm, “d4”: 2.5–3 cm. (TIF) [file pone.0192391.s001.tif]

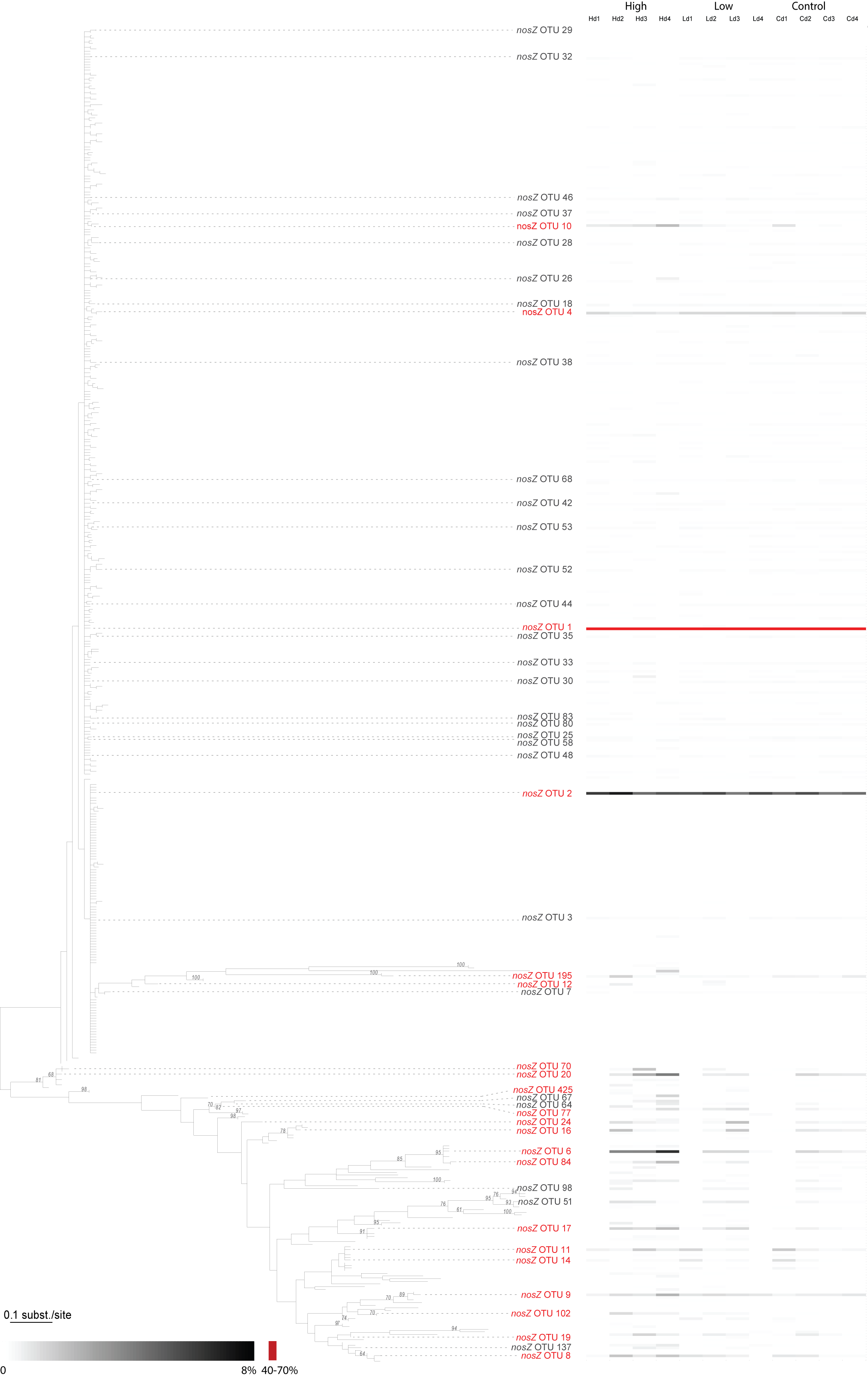

Supplement: S2 Fig — 10% of OTUs (50 OTUs out of 502) making up around 84% of the total reads are shown. Abundant OTUs (>1% relative abundance in at least one treatment–depth combination) are indicated in red. The heat map (on the right) illustrates the average relative abundance of each OTU per sample. The dominant OTU 1 is shown in red. Numbers at nodes are bootstrap values (values <60% not shown). The scale bar represents 10% sequence divergence (10 mutations per 100 sequence positions). (TIF) [file pone.0192391.s002.tif]

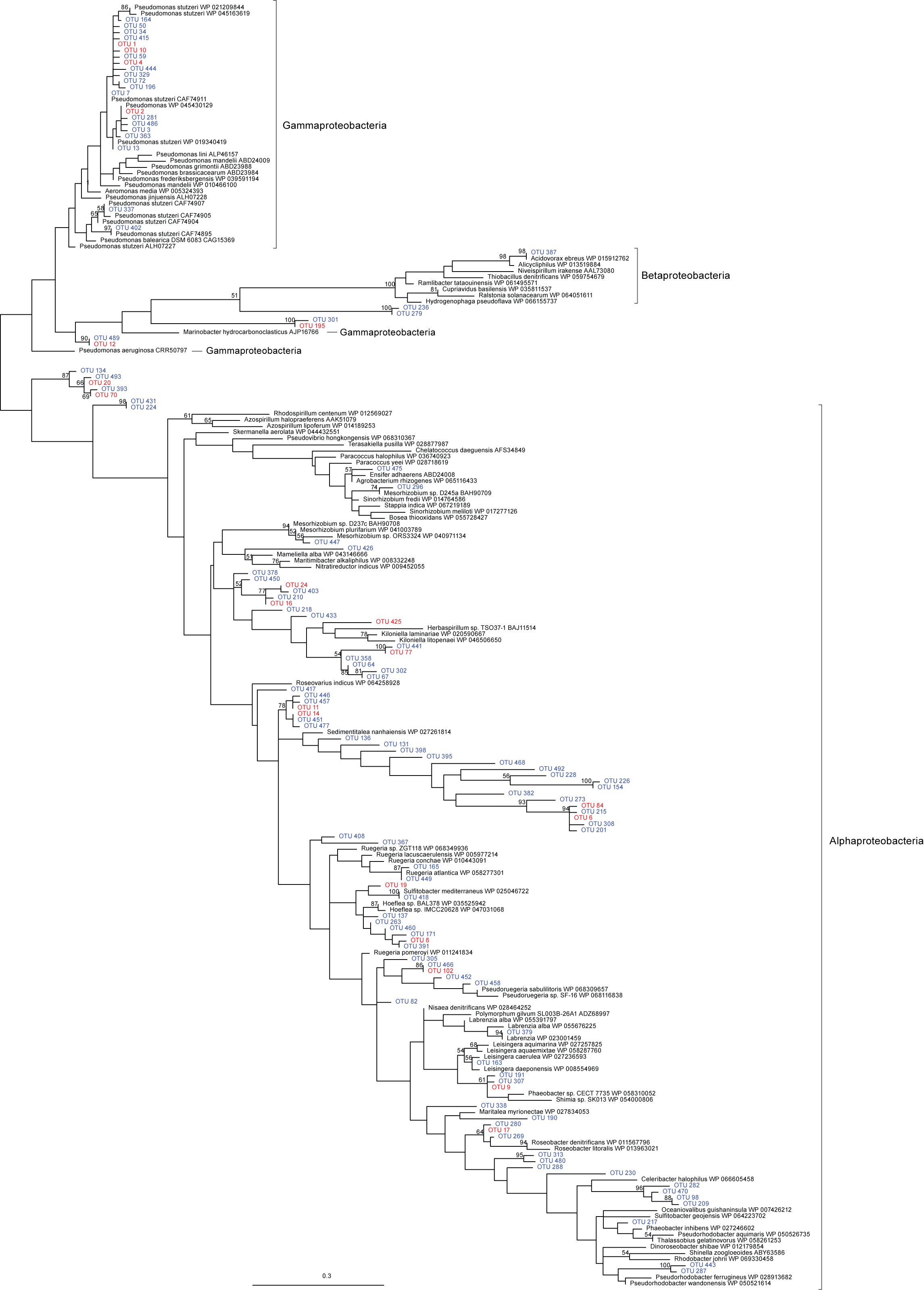

Supplement: S3 Fig — To construct a phylogeny with reference sequences, protein BLAST searches of 25 OTU representatives selected randomly across the phylogenetic tree in S2 Fig were performed against the NCBI non-redundant protein database. Abundant OTUs (>1% relative abundance) are shown in red. Numbers at nodes are bootstrap values (values <50% not shown). The scale bar represents 30% sequence divergence (30 mutations per 100 sequence positions). (TIF) [file pone.0192391.s003.tif]

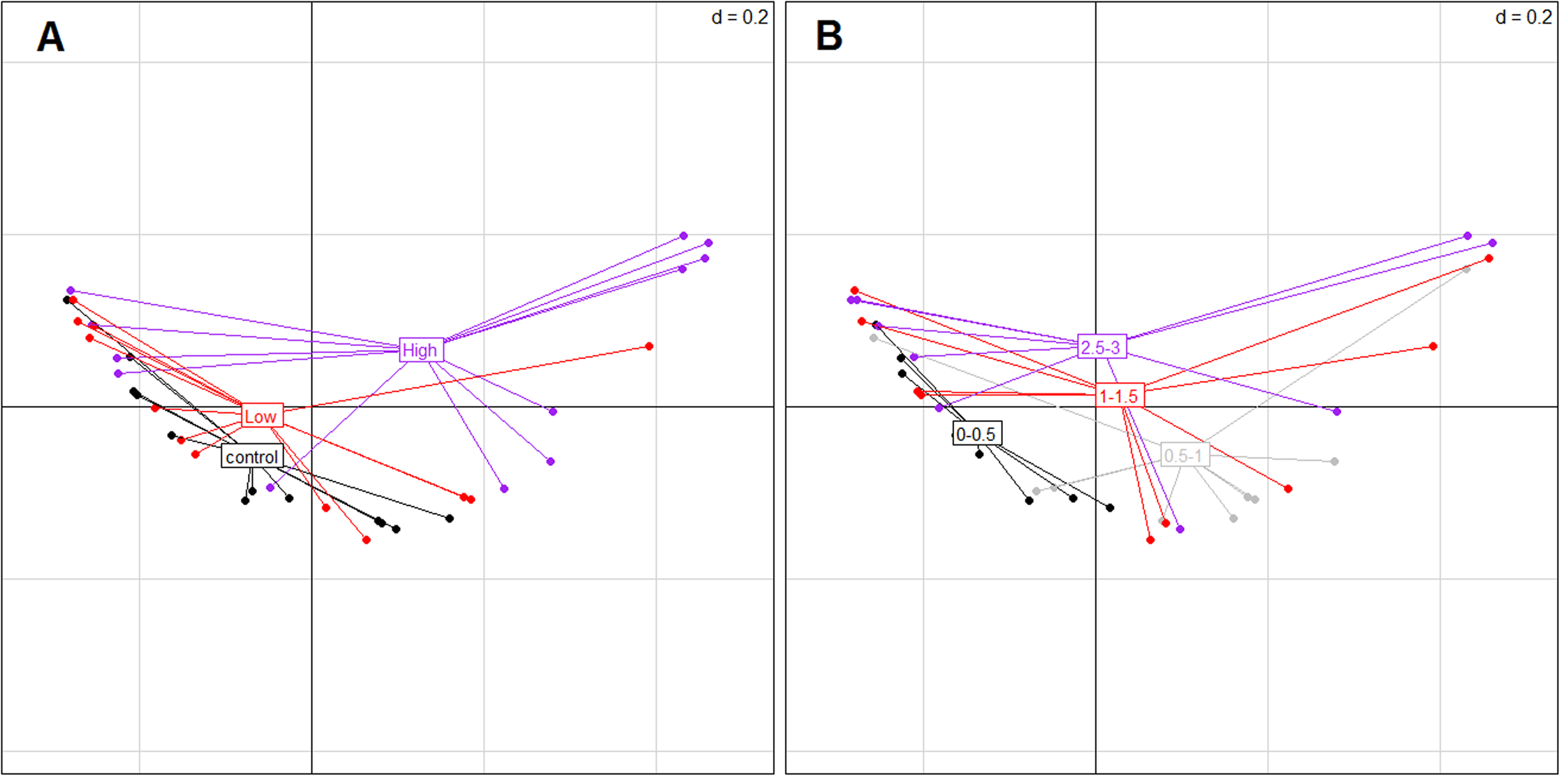

Supplement: S4 Fig — A: per treatment; B: per depth. Each point represents a sample. Three treatments (High: high L. conchilega treatment, Low: low L. conchilega treatment, Control) and four depth layers (0–0.5, 0.5–1, 1–1.5 and 2.5–3 cm). (TIF) [file pone.0192391.s004.tif]
