# Supplementary material for: The effect of bio-irrigation by the polychaete Lanice conchilega on active denitrifiers: Distribution, diversity and composition of nosZ gene
Source: PLoS One. 2018 Feb 6;13(2):e0192391. doi: 10.1371/journal.pone.0192391 (PMC5800672; doi:10.1371/journal.pone.0192391)
Supplement: S1 Table — Adaptor, Multiple Identifier (MID), pad, linker and primer sequences for the forward and reverse data sets are given. (DOCX) [file pone.0192391.s005.docx]

**S1 Table. Primers for paired-end *nosZ* sequencing on the Illumina Miseq platform.**

| ***FORWRD PRIMER*** |  |  |  |  |
| --- | --- | --- | --- | --- |
| *5' Illumina Adapter* |  | *Pad* | *Linker* | *Forward Primer (nosZ-2F)* |
| AATGATACGGCGACCACCGAGATCTACAC |  | TATGGTAATT | GT | CGCRACGGCAASAAGGTSMSSGT |
| ***REVERSE PRIMER*** |  |  |  |  |
| *Reverse complement of 3' Illumina Adapter* | *MID* | *Pad* | *Linker* | *Reverse primer (nosZ-2R)* |
| CAAGCAGAAGACGGCATACGAGAT | TACCGCTTCTTC | AGTCAGTCAG | CC | CAKRTGCAKSGCRTGGCAGAA |
| CAAGCAGAAGACGGCATACGAGAT | TGTGCGATAACA | AGTCAGTCAG | CC | CAKRTGCAKSGCRTGGCAGAA |
| CAAGCAGAAGACGGCATACGAGAT | GATTATCGACGA | AGTCAGTCAG | CC | CAKRTGCAKSGCRTGGCAGAA |
| CAAGCAGAAGACGGCATACGAGAT | GCCTAGCCCAAT | AGTCAGTCAG | CC | CAKRTGCAKSGCRTGGCAGAA |
| CAAGCAGAAGACGGCATACGAGAT | GATGTATGTGGT | AGTCAGTCAG | CC | CAKRTGCAKSGCRTGGCAGAA |
| CAAGCAGAAGACGGCATACGAGAT | ACTCCTTGTGTT | AGTCAGTCAG | CC | CAKRTGCAKSGCRTGGCAGAA |
| CAAGCAGAAGACGGCATACGAGAT | GTCACGGACATT | AGTCAGTCAG | CC | CAKRTGCAKSGCRTGGCAGAA |
| CAAGCAGAAGACGGCATACGAGAT | GCGAGCGAAGTA | AGTCAGTCAG | CC | CAKRTGCAKSGCRTGGCAGAA |
| CAAGCAGAAGACGGCATACGAGAT | ATCTACCGAAGC | AGTCAGTCAG | CC | CAKRTGCAKSGCRTGGCAGAA |
| CAAGCAGAAGACGGCATACGAGAT | ACTTGGTGTAAG | AGTCAGTCAG | CC | CAKRTGCAKSGCRTGGCAGAA |
| CAAGCAGAAGACGGCATACGAGAT | TCTTGGAGGTCA | AGTCAGTCAG | CC | CAKRTGCAKSGCRTGGCAGAA |
| CAAGCAGAAGACGGCATACGAGAT | TCACCTCCTTGT | AGTCAGTCAG | CC | CAKRTGCAKSGCRTGGCAGAA |
| CAAGCAGAAGACGGCATACGAGAT | GCACACCTGATA | AGTCAGTCAG | CC | CAKRTGCAKSGCRTGGCAGAA |
| CAAGCAGAAGACGGCATACGAGAT | GCGACAATTACA | AGTCAGTCAG | CC | CAKRTGCAKSGCRTGGCAGAA |
| CAAGCAGAAGACGGCATACGAGAT | TCATGCTCCATT | AGTCAGTCAG | CC | CAKRTGCAKSGCRTGGCAGAA |
| CAAGCAGAAGACGGCATACGAGAT | AGCTGTCAAGCT | AGTCAGTCAG | CC | CAKRTGCAKSGCRTGGCAGAA |
| CAAGCAGAAGACGGCATACGAGAT | GAGAGCAACAGA | AGTCAGTCAG | CC | CAKRTGCAKSGCRTGGCAGAA |
| CAAGCAGAAGACGGCATACGAGAT | TACTCGGGAACT | AGTCAGTCAG | CC | CAKRTGCAKSGCRTGGCAGAA |
| CAAGCAGAAGACGGCATACGAGAT | CGTGCTTAGGCT | AGTCAGTCAG | CC | CAKRTGCAKSGCRTGGCAGAA |
| CAAGCAGAAGACGGCATACGAGAT | TACCGAAGGTAT | AGTCAGTCAG | CC | CAKRTGCAKSGCRTGGCAGAA |
| CAAGCAGAAGACGGCATACGAGAT | CACTCATCATTC | AGTCAGTCAG | CC | CAKRTGCAKSGCRTGGCAGAA |
| CAAGCAGAAGACGGCATACGAGAT | GTATTTCGGACG | AGTCAGTCAG | CC | CAKRTGCAKSGCRTGGCAGAA |
| CAAGCAGAAGACGGCATACGAGAT | TATCTATCCTGC | AGTCAGTCAG | CC | CAKRTGCAKSGCRTGGCAGAA |
| CAAGCAGAAGACGGCATACGAGAT | TTGCCAAGAGTC | AGTCAGTCAG | CC | CAKRTGCAKSGCRTGGCAGAA |
| CAAGCAGAAGACGGCATACGAGAT | AGTAGCGGAAGA | AGTCAGTCAG | CC | CAKRTGCAKSGCRTGGCAGAA |
| CAAGCAGAAGACGGCATACGAGAT | GCAATTAGGTAC | AGTCAGTCAG | CC | CAKRTGCAKSGCRTGGCAGAA |
| CAAGCAGAAGACGGCATACGAGAT | CATACCGTGAGT | AGTCAGTCAG | CC | CAKRTGCAKSGCRTGGCAGAA |
| CAAGCAGAAGACGGCATACGAGAT | ATGTGTGTAGAC | AGTCAGTCAG | CC | CAKRTGCAKSGCRTGGCAGAA |
| CAAGCAGAAGACGGCATACGAGAT | CCTGCGAAGTAT | AGTCAGTCAG | CC | CAKRTGCAKSGCRTGGCAGAA |
| CAAGCAGAAGACGGCATACGAGAT | TTCTCTCGACAT | AGTCAGTCAG | CC | CAKRTGCAKSGCRTGGCAGAA |
| CAAGCAGAAGACGGCATACGAGAT | GCTCTCCGTAGA | AGTCAGTCAG | CC | CAKRTGCAKSGCRTGGCAGAA |
| CAAGCAGAAGACGGCATACGAGAT | GTTAAGCTGACC | AGTCAGTCAG | CC | CAKRTGCAKSGCRTGGCAGAA |
| CAAGCAGAAGACGGCATACGAGAT | ATGCCATGCCGT | AGTCAGTCAG | CC | CAKRTGCAKSGCRTGGCAGAA |
| CAAGCAGAAGACGGCATACGAGAT | GACATTGTCACG | AGTCAGTCAG | CC | CAKRTGCAKSGCRTGGCAGAA |
| CAAGCAGAAGACGGCATACGAGAT | GCCAACAACCAT | AGTCAGTCAG | CC | CAKRTGCAKSGCRTGGCAGAA |
| CAAGCAGAAGACGGCATACGAGAT | ATCAGTACTAGG | AGTCAGTCAG | CC | CAKRTGCAKSGCRTGGCAGAA |
|  |  |  |  |  |

Adaptor, Multiple Identifier (MID), pad, linker and primer sequences for the forward and reverse data sets are given.
