# Supplementary material for: The effect of bio-irrigation by the polychaete Lanice conchilega on active denitrifiers: Distribution, diversity and composition of nosZ gene
Source: PLoS One. 2018 Feb 6;13(2):e0192391. doi: 10.1371/journal.pone.0192391 (PMC5800672; doi:10.1371/journal.pone.0192391)
Supplement: S2 Table — Treatments: high and low L. conchilega densities and control. Analyses were based on Euclidean distance similarity matrix. Where the treatment effect was significant, pairwise tests were performed within treatments. P-values were obtained by permutation and drawn from Monte-Carlo samplings (p(MC)) if the number of unique permutations was less than 100 [70]. Max OPD indicates maximum oxygen penetration depth. Bold values indicate significant differences at p < 0.05. (DOCX) [file pone.0192391.s006.docx]

**S2 Table. Results from PERMANOVA analysis for differences in O_2_ concentrations (depth and time profiles and max OPD) among treatments.**

| ***Main test*** |  |  |  |
| --- | --- | --- | --- |
|  | *factor* | *Pseudo-F* | *P_(perm)_* |
| *O_2_ depth profiles* | treatment | 5.33 | **0.006** |
| *Max OPD* | treatment | 12.19 | **0.000** |
| *O_2_ time profiles* | treatment | 2.11 | 0.178 |
| ***Pairwise test*** |  |  |  |
|  |  | *t* | *P_(perm)_* |
| *O_2_ depth profiles* | High – Low density | 1.36 | 0.168 |
|  | High density - Control | 3.39 | **0.002** |
|  | Low density - Control | 2.03 | **0.039** |
| *Max OPD* |  | *t* | *P_(MC)_* |
|  | High – Low density | 3.11 | **0.008** |
|  | High density - Control | 4.08 | **0.001** |
|  | Low density - Control | 1.88 | 0.079 |

Treatments: high and low *L. conchilega* densities and control. Analyses were based on Euclidean distance similarity matrix. Where the treatment effect was significant, pairwise tests were performed within treatments. P-values were obtained by permutation and drawn from Monte-Carlo samplings (p_(MC)_) if the number of unique permutations was less than 100 [70]. Max OPD indicates maximum oxygen penetration depth. Bold values indicate significant differences at p < 0.05.
