# Supplementary material for: The effect of bio-irrigation by the polychaete Lanice conchilega on active denitrifiers: Distribution, diversity and composition of nosZ gene
Source: PLoS One. 2018 Feb 6;13(2):e0192391. doi: 10.1371/journal.pone.0192391 (PMC5800672; doi:10.1371/journal.pone.0192391)
Supplement: S3 Table — (DOCX) [file pone.0192391.s007.docx]

**S3 Table. Numbers of reads and total numbers of OTUs per sample as well as the numbers of abundant OTUs (>1% relative abundance) in treatment-depth combinations are provided.**

|  | Number of Reads | Number of  total OTUs | Number of  abundant (> 1%) OTUs |
| --- | --- | --- | --- |
| h1d1 | 6,945 | 296 | Hd1=3 |
| h2d1 | 16,296 | 368 |  |
| h3d1 | 31,954 | 425 |  |
| h1d2 | 5,639 | 284 | Hd2=10 |
| h2d2 | 3,007 | 220 |  |
| h3d2 | 1,322 | 114 |  |
| h1d3 | 4,741 | 250 | Hd3=10 |
| h2d3 | 1,455 | 121 |  |
| h3d3 | 14,570 | 373 |  |
| h1d4 | 1,242 | 123 | Hd4=12 |
| h2d4 | 1,022 | 104 |  |
| h3d4 | 1,956 | 162 |  |
| L1d1 | 9,051 | 320 | Ld1=5 |
| L2d1 | 38,734 | 449 |  |
| L3d1 | 8,485 | 315 |  |
| L1d2 | 4,003 | 245 | Ld2=5 |
| L2d2 | 25,073 | 402 |  |
| L3d2 | 7,652 | 316 |  |
| L1d3 | 1,143 | 128 | Ld3=8 |
| L2d3 | 15,863 | 348 |  |
| L3d3 | 7,194 | 290 |  |
| L1d4 | 24,498 | 416 | Ld4=3 |
| L2d4 | 33,194 | 406 |  |
| L3d4 | 20,627 | 349 |  |
| C1d1 | 15,082 | 343 | Cd1=5 |
| C2d1 | 38,098 | 439 |  |
| C3d1 | 28,538 | 430 |  |
| C1d2 | 12,906 | 357 | Cd2=5 |
| C2d2 | 13,119 | 363 |  |
| C3d2 | 15,272 | 374 |  |
| C1d3 | 15,166 | 381 | Cd3=3 |
| C2d3 | 14,870 | 377 |  |
| C3d3 | 29,275 | 416 |  |
| C1d4 | 31,399 | 400 | Cd4=3 |
| C2d4 | 13,554 | 361 |  |
| C3d4 | 32,797 | 388 |  |
