# Supplementary material for: The effect of bio-irrigation by the polychaete Lanice conchilega on active denitrifiers: Distribution, diversity and composition of nosZ gene
Source: PLoS One. 2018 Feb 6;13(2):e0192391. doi: 10.1371/journal.pone.0192391 (PMC5800672; doi:10.1371/journal.pone.0192391)
Supplement: S4 Table — Analyses were carried out based on Generalized UniFrac distances (α = 0.5) on the data sets using all OTUs or only abundant OTUs with relative abundance >1%. Treatments: high and low L. conchilega densities and control; Depths: 0–0.5, 0.5–1, 1–1.5 and 2.5–3 cm. (DOCX) [file pone.0192391.s008.docx]

**S4 Table. Results from PERMANOVA analysis pairwise tests for differences in composition of *nosZ* influenced by the single effects of “treatment” and “depth”.**

| ***All OTUs*** | |  |  |  |  |  |  |
| --- | --- | --- | --- | --- | --- | --- | --- |
|  |  |  |  |  |  |  |  |
| *Treatments* |  | **High / Low** | **High / Control** | **Low / Control** |  |  |  |
|  | Pseudo-F | 2.33 | 3.23 | 0.79 |  |  |  |
|  | P value | 0.058 | **0.036** | 0.455 |  |  |  |
| *Depth (cm)* |  | **0-0.5 / 0.5-1** | **0-0.5 / 1-1.5** | **0-0.5 / 2.5-3** | **0.5-1 /1-1.5** | **0.5-1 / 2.5-3** | **1-1.5 / 2.5-3** |
|  | Pseudo-F | 7.63 | 3.45 | 3.37 | 0.75 | 1.64 | 1.51 |
|  | P value | **0.001** | **0.018** | **0.020** | 0.476 | 0.168 | 0.640 |
| ***Abundant (> 1%) OTUs*** | | |  |  |  |  |  |
|  |  |  |  |  |  |  |  |
| *Treatments* |  | **High / Low** | **High / Control** | **Low / Control** |  |  |  |
|  | Pseudo-F | 2.64 | 3.96 | 0.51 |  |  |  |
|  | P value | 0.077 | **0.034** | 0.608 |  |  |  |
| *Depth (cm)* |  | **0-0.5 / 0.5-1** | **0-0.5 / 1-1.5** | **0-0.5 / 2.5-3** | **0.5-1 /1-1.5** | **0.5-1 / 2.5-3** | **1-1.5 / 2.5-3** |
|  | Pseudo-F | 7.46 | 3.23 | 3.70 | 0.66 | 1.35 | 0.33 |
|  | P value | **0.004** | **0.045** | **0.040** | 0.506 | 0.266 | 0.712 |

Analyses were carried out based on Generalized UniFrac distances (α = 0.5) on the data sets using all OTUs or only abundant OTUs with relative abundance > 1%. Treatments: high and low *L.conchilega* densities and control; Depths: 0-0.5, 0.5-1, 1-1.5 and 2.5-3 cm
