# Supplementary material for: The effect of bio-irrigation by the polychaete Lanice conchilega on active denitrifiers: Distribution, diversity and composition of nosZ gene
Source: PLoS One. 2018 Feb 6;13(2):e0192391. doi: 10.1371/journal.pone.0192391 (PMC5800672; doi:10.1371/journal.pone.0192391)
Supplement: S5 Table — Treatments: high and low L. conchilega densities and control; depths: 0–0.5, 0.5–1, 1–1.5 and 2.5–3 cm. Diversity indices (richness, Shannon-Wiener [log e] and inverse Simpson) were calculated from the average values obtained from 1000 sub-samples of the data matrix to the minimum number of reads (1022). Univariate analyses of diversity indices were based on Euclidean distance similarity matrix. P-values obtained by permutation. (DOCX) [file pone.0192391.s009.docx]

**S5 Table. Results from PERMANOVA analysis main tests for differences in diversity indices of *nosZ* among treatments and depths.**

|  | *factor** | *df_term_* | *Pseudo-F* | *P* |
| --- | --- | --- | --- | --- |
|  |  |  |  |  |
| ***Richness*** | treatment x depth | 6 | 5.45 | **0.043** |
| ***Shannon diversity*** | - | - | - | No sig. ** |
| ***Inverse Simpson*** | - | - |  | No sig. ** |
| *** indicates only significant factors  **p > 0.05 | | | | |

Treatments: high and low *L.conchilega* densities and control; depths: 0-0.5, 0.5-1, 1-1.5 and 2.5-3 cm. Diversity indices (richness, Shannon-Wiener [log e] and inverse Simpson) were calculated from the average values obtained from 1000 sub-samples of the data matrix to the minimum number of reads (1022). Univariate analyses of diversity indices were based on Euclidean distance similarity matrix. P-values obtained by permutation.
