# Supplementary material for: The effect of bio-irrigation by the polychaete Lanice conchilega on active denitrifiers: Distribution, diversity and composition of nosZ gene
Source: PLoS One. 2018 Feb 6;13(2):e0192391. doi: 10.1371/journal.pone.0192391 (PMC5800672; doi:10.1371/journal.pone.0192391)
Supplement: S6 Table — Three treatments (High: high L. conchilega treatment, Low: low L. conchilega treatment, Control) and four depths (0–0.5, 0.5–1, 1–1.5 and 2.5–3 cm). Richness was calculated from the average values obtained from 1000 sub-samples of the data matrix to the minimum number of reads (1022). Analyses were based on Euclidean distance similarity matrix. P-values obtained by permutation. (DOCX) [file pone.0192391.s010.docx]

**S6 Table. Results from PERMANOVA analysis pairwise tests for differences in OTU richness influenced by the interaction effect “treatment x depth”.**

| *Treatments* | | **High /**  **Low** | **High / Control** | **Low / Control** |  |  |  |
| --- | --- | --- | --- | --- | --- | --- | --- |
|  | |  |  |  |  |  |  |
| **0-0.5 *cm*** | t | 2.57 | 2.13 | 0.55 |  |  |  |
|  | P_(MC)_ | 0.624 | 0.105 | 0.608 |  |  |  |
| **0.5-1 *cm*** | t | 0.92 | 3.49 | 1.65 |  |  |  |
|  | P_(MC)_ | 0.408 | **0.026** | 0.182 |  |  |  |
| **1-1.5 *cm*** | t | 0.19 | 2.11 | 2.44 |  |  |  |
|  | P_(MC)_ | 0.859 | 0.098 | 0.072 |  |  |  |
| **2.5-3 *cm*** | t | 3.99 | 4.13 | 0.28 |  |  |  |
|  | P_(MC)_ | **0.016** | **0.015** | 0.795 |  |  |  |
| *Depth (cm)* | | **0-0.5 / 0.5-1** | **0-0.5 / 1-1.5** | **0-0.5 / 2.5-3** | **0.5-1 /1-1.5** | **0.5-1 / 2.5-3** | **1-1.5 / 2.5-3** |
|  | |  |  |  |  |  |  |
| **High** | t | 0.68 | 1.39 | 6.30 | 0.15 | 1.06 | 2.87 |
|  | P_(MC)_ | 0.570 | 0.294 | **0.022** | 0.894 | 0.403 | 0.104 |
| **Low** | t | 1.74 | 2.50 | 0.94 | 1.60 | 0.31 | 0.93 |
|  | P_(MC)_ | 0.220 | 0.128 | 0.452 | 0.244 | 0.783 | 0.462 |
| **Control** | t | 2.49 | 0.42 | 2.93 | 0.79 | 2.29 | 1.76 |
|  | P_(MC)_ | 0.137 | 0.729 | 0.103 | 0.513 | 0.124 | 0.145 |

Three treatments (High: high *L. conchilega* treatment, Low: low *L. conchilega* treatment, Control) and four depths (0-0.5, 0.5-1, 1-1.5 and 2.5-3 cm). Richness was calculated from the average values obtained from 1000 sub-samples of the data matrix to the minimum number of reads (1022). Analyses were based on Euclidean distance similarity matrix. P-values obtained by permutation.
